# Self‐Navigated, Retrospective, Data‐Consistent Motion Correction for MPnRAGE

**DOI:** 10.1002/mrm.70126

**Published:** 2025-10-13

**Authors:** John Podczerwinski, Andrew L. Alexander, Brittany G. Travers, James J. Li, Steven R. Kecskemeti

**Affiliations:** ^1^ Waisman Center University of Wisconsin‐Madison Madison Wisconsin USA; ^2^ Department of Medical Physics University of Wisconsin‐Madison Madison Wisconsin USA; ^3^ Department of Psychiatry University of Wisconsin‐Madison Madison Wisconsin USA; ^4^ Department of Kinesiology University of Wisconsin‐Madison Madison Wisconsin USA; ^5^ Department of Psychology University of Wisconsin‐Madison Madison Wisconsin USA

**Keywords:** k‐space, motion correction, MPnRAGE, radial, T1‐weighted

## Abstract

**Purpose:**

To extend and automate a data‐consistent, self‐navigated motion‐correction method for 3D radial T1‐weighted imaging.

**Methods:**

This method incorporated rigid‐body motion effects into the forward model, solving for parameters that maximize consistency with the data. The method was tested on five datasets with a range of motion types and severities. A separate collection of datasets was used to study the effect that the method has on the test‐retest reliability of cortical thickness estimates.

**Results:**

Image quality was improved across a wide range of distinct motion types, including some cases that would have been unusable if left uncorrected. The error‐based weighting scheme and the increased timing resolution afforded by the proposed method were especially useful in cases of extreme and rapid motions. Moreover, the method improved test‐retest reliability of cortical thickness measures in pediatric subjects, decreasing the average coefficient of variation from 2.73%±1.75% in uncorrected images (with freesurfer failing on one subject) down to 0.88%±0.21% for images corrected at ∼2s timing resolution and 0.79%±0.16% when corrected at faster temporal rates.

**Conclusion:**

This method was found to be effective when used on T1‐weighted radial data, both qualitatively and quantitatively. The fine‐scale timing resolution and error‐based weighting afforded by this technique will likely provide only a small benefit, unless one is investigating motion‐prone populations or is searching for a very small effect size.

## 1 Introduction

In conventional MR imaging with Cartesian k‐space trajectories, bulk subject motion produces overlapping ghosting artifacts that often hinder diagnostic ability. On the other hand, imaging with radial k‐space trajectories produces a blurring artifact when motion is present [1]. In the context of neuroimaging research, these motion effects have been noted to increase errors in estimates of cortical volume, thickness, surface area, and R1 [2], which can hinder group analysis if group‐level differences in motion occur.

Prospective motion correction accounts for rigid‐body motion by prospectively adjusting the readout gradient during the acquisition. One may estimate motion by optically tracking the subject's head using applied visual markers [3] or by tracking facial features or fiducials [4]. Such approaches have been shown to be effective, and are notable for providing fine timing resolution, with one paper showing a timing resolution of 10ms [5]. The drawback to these optical tracking methods is that they require expensive hardware, are potentially sensitive to facial movements not described by the assumption of rigid‐body motion, and require an unobstructed line of sight between the camera and the subject. Another prospective correction technique works by taking intermittent and dedicated “navigator” scans [6]. This approach often increases scan duration and assumes that there are periods of time that are motion‐free.

Alternatively, retrospective techniques perform motion correction during the image reconstruction process, usually with adjusted k‐space coordinates and phase shifts. In some cases, these techniques are also self‐navigated, meaning that they extract motion estimates directly from the same data used for imaging. Thus, they do not require specialized hardware or sequence modifications.

One retrospective approach is to estimate rigid‐body motion from low‐resolution navigator images reconstructed from subsets of the imaging data [7] acquired with 3D radial k‐space trajectories. This approach was shown to effectively correct for motion at two‐second timing resolution without the need for advanced image reconstruction strategies.

There are, of course, many more motion estimation methods for retrospective motion correction on 3D radial data. One approach is to use spatial information from coils along with “FIDnavs”, which are navigators generated from the center of k‐space [8, 9]. This method is promising in that it would, in principle, allow one to estimate motion on a view‐by‐view basis, although it is less robust against increased motions. One paper showed errors becoming significant for motions greater than 10mm [9], and another showed this method struggling with large “abrupt” motions [8]. Another paper [10] estimated motion by projecting the center of mass (COM) from each coil onto k‐space spokes. This method was shown to provide good results, but required special receiver coils that remain fixed to the head. A navigator‐based motion‐correction method has also been introduced for 3D radial T2‐weighted imaging [11]. In this method, motion is estimated by registering navigator images formed from subsets of the data. In another approach, motion events were identified by tracking the COM as a function of time [12]. Data was then binned according to motion state, reconstructed, and co‐registered. This approach worked well for step‐like motions, but performed poorly on drifting motions.

In this paper, motion is accounted for by putting a rigid‐body motion operator in the forward signal model. This sort of approach has been proposed in earlier papers [13, 14]. In one of these papers [14], a joint optimization problem is proposed to solve for the underlying image and rigid‐body motion parameters while enforcing data fidelity with an L2 penalty to ensure k‐space consistency of the final image. An accelerated version that uses a scout scan for motion estimation has also been proposed [15, 16]. In the first paper [14] and a notable follow‐up manuscript [17], the emphasis has been on Cartesian imaging, with promising results demonstrating that correction is possible for both large and rapid motions, without the need for additional hardware or navigator scans. These works also illustrated the importance of having a sampling pattern that prevents rotations from causing gaps in k‐space. For instance, it was shown that sequential k‐space sampling did not support large decreases in the objective function and that random Cartesian sampling was unable to handle rotations larger than $20^{\circ }$, with these limitations being due to the creation of k‐space gaps [14]. Moreover, a test using sequential radial sampling failed to minimize the objective function for rotations larger than $2^{\circ }$. However, a later paper [17] introduced a novel Cartesian sampling pattern to alleviate this issue. In this work, we do something similar by pairing such an approach with a 3D radial sampling pattern that is robust to rotations. This pairing allows for the correction of large and rapid motions without the need for additional hardware or navigator scans.

In this work, we extend this motion‐correction approach to MPnRAGE. The MPnRAGE method utilizes inversion recovery and 3D radial k‐space sampling to image a large number, n, of T1‐weighted images during the inversion recovery. To efficiently handle the increased computation costs of a dynamic signal curve across the n inversion times, a low‐rank subspace approach [18] is applied. Similar subspace‐based approaches are used in spectroscopic and dynamic imaging [19]. The pseudo‐random sampling pattern of MPnRAGE also naturally permits retrospective selection of the temporal rate of motion estimation and protects against unsampled regions of k‐space. An automated method based on image sharpness is used to select the optimal rate. A method to suppress high error data is also introduced.

## 2 Methods

### 2.1 Acquisition

**FIGURE 1:**
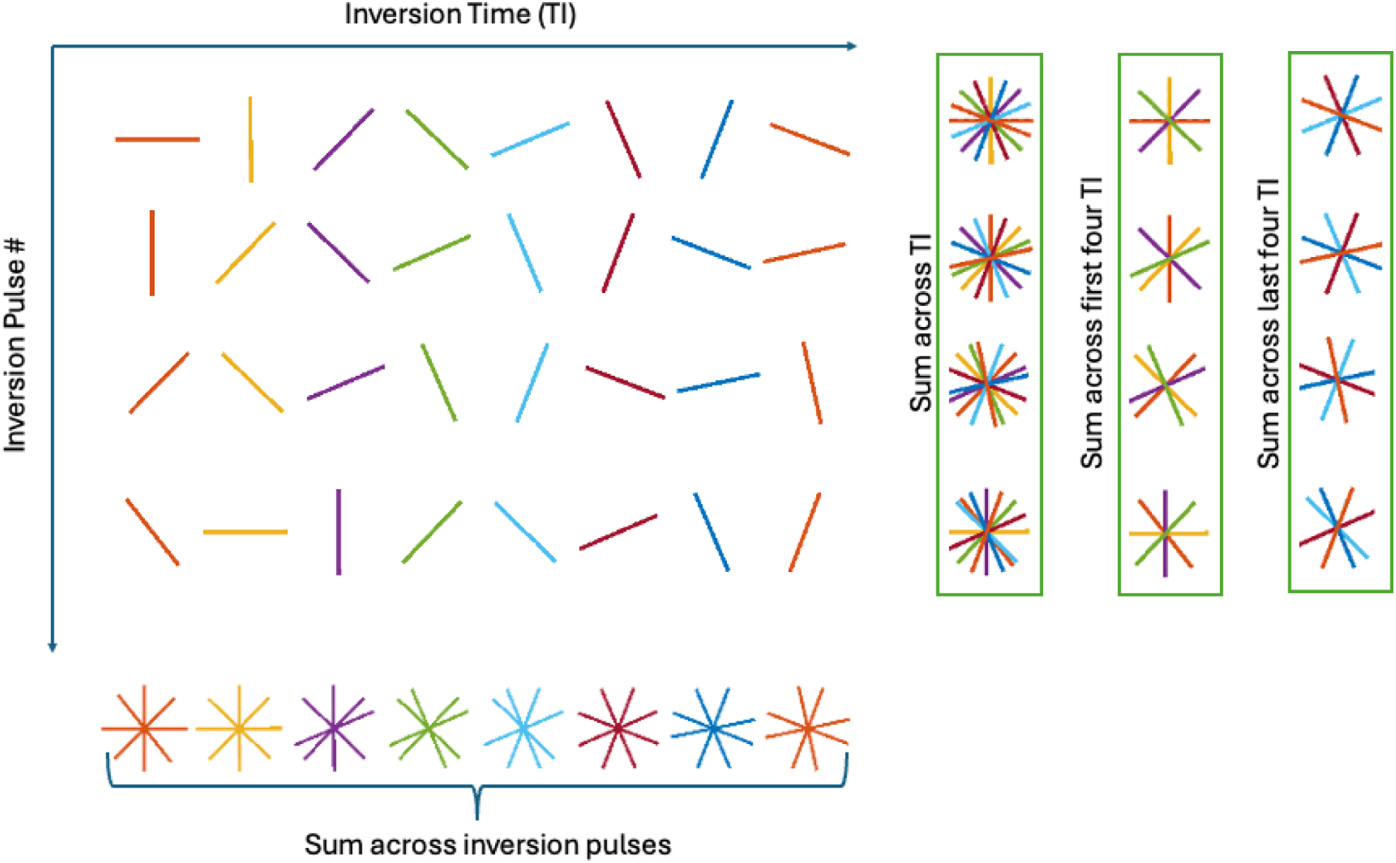
A 2D illustration of the double bit‐reversed sampling scheme used by MPnRAGE. The “Sum across inversion pulses” part of the figure illustrates how the first bit‐reversal ensures approximately uniform sampling across inversion pulses. The columns showing sums across TI illustrate how the second bit‐reversal ensures approximately uniform sampling across inversion times for all inversion pulses.

This work utilizes the MPnRAGE acquisition, which is an adaptation of the MPRAGE acquisition with 3D radial k‐space sampling. The view ordering is arranged using a double bit‐reversed acquisition to maintain pseudo‐random sampling both for all views between consecutive inversion pulses and for each given view across multiple inversion pulses. A 2D schematic of this sampling pattern is illustrated in Figure 1. The first bit‐reversal creates an acquisition order of views between inversion pulses that is uniformly distributed. This allows for retrospective selection of the temporal resolution of motion estimates. The second bit‐reversal arranges the views so that for an individual inversion time, views across inversion pulses (i.e., motion states) get approximately uniformly distributed. This creates a more isotropic blur in uncorrected images [1] and will reduce gaps in the actually sampled k‐space after motion correction is performed. This ordering has been used in previous MPnRAGE manuscripts [2, 7, 20].

### 2.2 Theory and Background

It has been shown that this reconstruction approach can be formulated as [14]

(1) $$(\hat{X},\hat{q})=\underset{q,X}{argmin}\|W^{1/2}(EM(q)X-d){\|}^{2}$$

In this formula, X is an image matrix with dimensions $N_{v}\times N_{t}$, where $N_{v}$ is the number of voxels and $N_{t}$ is the number of inversion times during the recovery. The operator M applies rigid‐body transformations to the image based on motion parameters q. For this work, rigid‐body transformations are performed by applying a series of shears and phases in k‐space [14]. The matrix E is the encoding matrix, which contains both coil sensitivities and a non‐uniform FFT. Lastly, W is a density compensation term [21].

In this method, the k‐space data are partitioned into subsets based on the time since the start of data acquisition. Translations and rotations are estimated for each subset to correct for rigid‐body motion. One natural partitioning scheme for T1‐weighted sequences is to estimate one set of rigid‐body parameters per inversion pulse. In this paper, the data collected after an inversion pulse are referred to as a “block”. However, the data may be partitioned more finely, into $N_{s}$ segments per block. When partitioning a block into segments, each segment is constructed to have an equal number of views. This concept is illustrated in Figure 1. In this example, the “sum across TI” column shows the data segments that would be used when correcting at $N_{s}=1$ segments per block. The “sum across first four TI” and “sum across last four TI” columns show the segments that would be used when correcting at $N_{s}=2$.

When using MPnRAGE, $N_{t}$ is typically on the order of hundreds. To save computational time, a low‐rank scheme is used to compress the image. This scheme takes advantage of the simple nature of the inversion recovery curve. In particular, the recovery curve depends only on a few variables, meaning that it is possible to describe it using a small number ($N_{b}$) of basis functions. These basis functions are determined a priori based on the full signal model, which takes into account $B_{1}$, inversion efficiency, and the expected range of $T_{1}$ values. More specifically, an array of simulated recovery curves is generated using random values of the relevant parameters. An SVD is then performed on this array, with the first $N_{b}$ right vectors being used as basis functions. It should be noted that this model assumes that there are no spin‐refocusing effects across views, as the MPnRAGE sequence includes RF and gradient spoiling. The accuracy of this assumption was verified in a previous work [22].

Let R be a matrix with dimensions $N_{b}\times N_{t}$ that contains these basis functions. Let L be a matrix with dimensions $N_{v}\times N_{b}$, which gives the appropriate mixture of basis functions at each voxel in the image. The image matrix X can then be expressed via

(2) X=LR

The problem of interest described in Equation (1) can then be solved in an iterative fashion. The images are updated by minimizing k‐space error while keeping the motion parameters (q) fixed:

(3) $$L^{n+1}=\underset{L}{argmin}\frac{1}{2}\|W^{1/2}(EM(q^{n})LR-d){\|}^{2}$$

Similarly, q is updated while L is kept fixed

(4) $$\begin{array}{l} q^{n+1}=\underset{q}{argmin}\|W^{1/2}(EM(q)L^{n+1}R-d){\|}^{2} \end{array}$$

### 2.3 The Weighting Scheme

In some cases, one will find that the method struggles to appropriately correct some blocks of a dataset, while other blocks are better corrected. Some examples of causes for these variations are within‐block motions and changes in coverage between blocks (e.g., shoulders going in and out of the FOV). The poorly corrected blocks can be suppressed using an error‐based weighting scheme. In particular, Equation (3) can be reformulated as

(5) $$L^{n+1}=\underset{L}{argmin}\frac{1}{2}\|{\left(G^{\left(n\right)}\right)}^{1/2}W^{1/2}(EM(q^{n})LR-d){\|}^{2}$$

In this formula, G is a block‐by‐block weighting term. The weight for block i is updated via:

(6) $$G_{i}^{n+1}=\frac{1}{\|{\left(EM(q^{n+1})L^{n+1}R\right)}_{i}-d_{i}{\|}^{2}}$$

The effect of this weighting scheme is as follows: In regions of k‐space that are oversampled, the weights will favor better corrected blocks, giving them a larger contribution to the resulting images at such scales. It should be noted that oversampled k‐space regions are inevitable in radial imaging sequences, where low‐k values are measured repeatedly.

The higher error data are downweighted, rather than excluded, for several reasons. For one, applying a weighting scheme increases the sharpness of images with less detriment to SNR or risk of artifacts [23]. Moreover, using a weighting scheme allows for the process to be automated without having to choose some arbitrary error cutoff.

The weighting is done on a block‐by‐block basis in order to avoid perturbing the contrast of the resulting images. The $L_{2}$ norm was chosen based on preliminary tests. Though not shown here, it was found that using the square of the error provided sharper images compared to those generated using the $L_{1}$ norm weighting or the square root of the $L_{2}$ norm. One may refer to Figures S28–S45 in the supplement document to see example images from these tests.

### 2.4 Implementation Details

**FIGURE 2:**
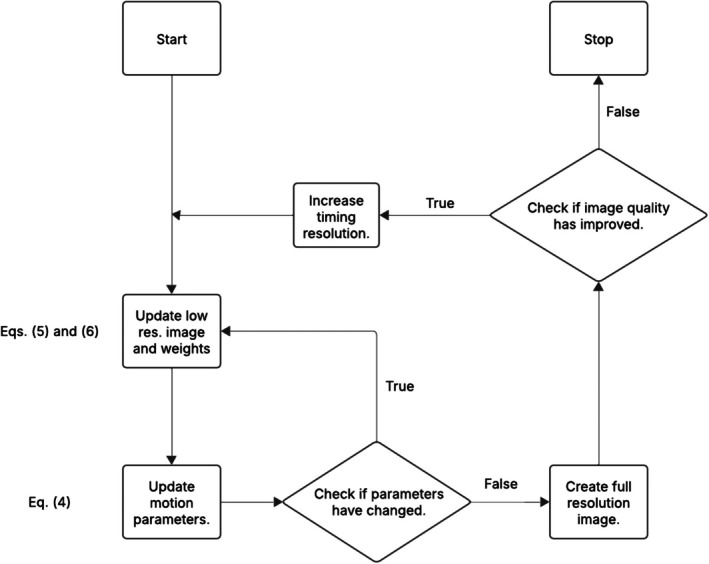
A flow chart giving a simplified description of the motion‐correction process. Rectangles represent processes and diamonds represent decisions.

A flow chart giving a simplified description of the approach can be seen in Figure 2.

The approach begins by iteratively solving Equation (4), (5) and (6), obtaining motion estimates at $N_{s}=1$ segments per block. In this paper, motion parameters (q) are updated using the Levenberg‐Marquardt (LM) technique, and images (L) are updated using the Conjugate Gradient (CG) method. This optimization is done using low‐resolution (4mm) images formed from the data. This lower spatial resolution is used to save on computation time and was found to have little impact on the resulting motion estimates.

After obtaining motion estimates, full resolution (1mm) images are reconstructed. The sharpness of the high‐resolution images is quantified using the Tenegrad Measure (TM) [24]. For the experiments described in this paper, anything greater than a 0.1% increase in TM is considered to be a non‐negligible improvement.

If the newly estimated parameters are found to significantly improve TM, then the timing resolution parameter $N_{s}$ is increased and the process is repeated.

When increasing timing resolution, it is important to initialize the motion estimation processing using the images or motion parameters obtained from the previous, lower timing resolution. This helps the solver avoid local minima caused by decreasing SNR.

### 2.5 Simulated Experiment

An additional simulated experiment was performed to test the accuracy of parameter estimates. For this experiment, a set of motion parameters was generated randomly. For each inversion recovery block, rotations along each axis were drawn from a Gaussian distribution with a standard deviation of $\sigma_{rot}=3^{\circ }$ and translations along each axis were drawn from a distribution with $\sigma_{trans}=3\;\text{mm}$. These motions were then applied to an image with very little motion borrowed from Section 2.7. These motion‐affected images were then used to generate a motion‐affected k‐space data. The motion‐correction method was then used to estimate the motion parameters from these simulated data.

### 2.6 Initial In‐Vivo Experiments

Imaging experiments were performed with institutional review board approval and after informed consent/assent. All imaging took place on a 3T MR750 Scanner (GE Healthcare, Waukesha, WI) and utilized a 32‐channel receive‐only phased array coil from Nova Medical (Wilmington, MA).

Initial tests are performed on images of five subjects with various levels and types of motion. These images were taken from a study of children diagnosed with ASD, ADHD, or both. For the cases considered, the subjects were between ages 6 and 8 *years*.

All imaging was performed using the MPnRAGE sequence at isotropic 1.0mm resolution over a 256×256×256mm FOV. This sequence had parameters TR=4.9ms, TE=1.8ms, and a time of TD=500ms between blocks for signal recovery. There were 225 blocks. Each block contained a total of 386 views: 305(81) views with flip angle $4^{\circ }$($8^{\circ }$).

### 2.7 In‐Vivo Cortical Thickness Experiments

Twelve typically developing children between 6.5 years and 13.8 years (mean +/‐ std = 9.4±2.6years) were scanned three times over the course of a one‐hour imaging session.

The scans were taken using MPnRAGE with mostly the same resolution and timing parameters as given in Section 2.4. The main difference is that these datasets have 200 slices along the axial direction, with 256×256mm in plane. These datasets contain 176 blocks. Each block has 325(61) views with flip angle $4^{\circ }(8^{\circ })$.

For this test, three CG‐refined full‐resolution images are generated for each subject and time point. One without motion correction, one corrected at $N_{s}=1$ segments per block, and one corrected at the timing resolution that provided the highest TM value. The motion‐corrected images are reconstructed using the weighting scheme. For each of these images, cortical thickness estimates are generated using the Freesurfer software package [25]. Estimates of average thickness are assessed for each region of the Destrieux atlas.

For each subject, timing resolution, and atlas label, the coefficient of variation (COV) is estimated across the three longitudinal time points available. For this analysis, hemispheric averaged values are used.

## 3 Results

### 3.1 Simulation Results

Motion parameter estimates were quite accurate. For rotations, the average error was $0.028^{\circ }$. The average error for translations was 0.017mm. Plots and reconstructed images from this example can be found in Figures S14–S27 in the supplement.

### 3.2 Motion Correction Versus Motion Type

**FIGURE 3:**
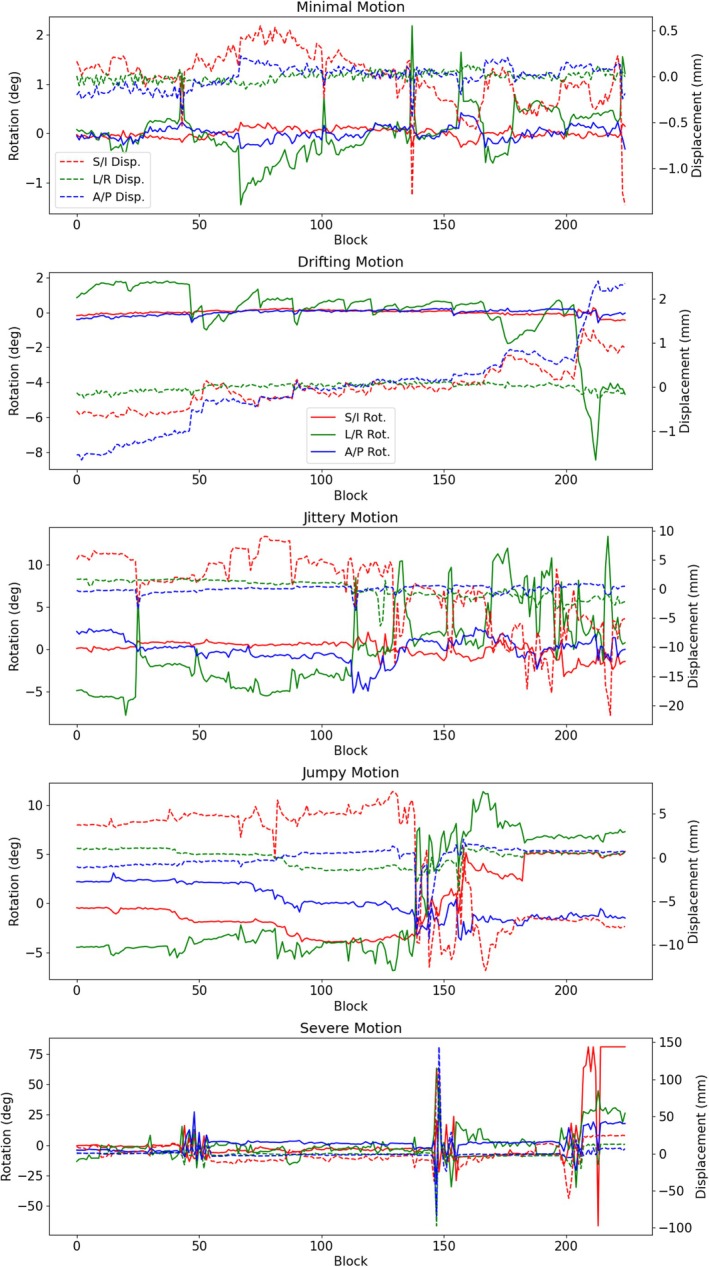
Plots of motion parameters for cases with different motion characteristics. Dashed lines indicate displacements and solid lines indicate rotations. Note that each case is scaled independently and that each block represents a roughly two‐second time window.

The motion parameters obtained for the five test cases are shown in Figure 3, and they are labeled as follows based on the types and degrees of motion:

1. **Minimal Motion:** Slight motion, with rotations on the order of about $1^{\circ }$ and translations <1mm.
2. **Drifting Motion:** Most motion was a gradual drift, mostly in the A/P direction.
3. **Jittery Motion:** Approximately 50% of the scan has rapid motions with rotations up to about $10^{\circ }$ and the displacements up to about 20mm.
4. **Jumpy Motion:** Motion was dominated by a large, severe “jump” in motion. During the jump, the angle on the L/R axis changes by about $10^{\circ }$ and the S/I displacement parameter changes by about 15mm.
5. **Severe Motion:** There are rapid motions throughout the scan and several events with rotations and translations up to tens of degrees and tens of millimeters.

**FIGURE 4:**
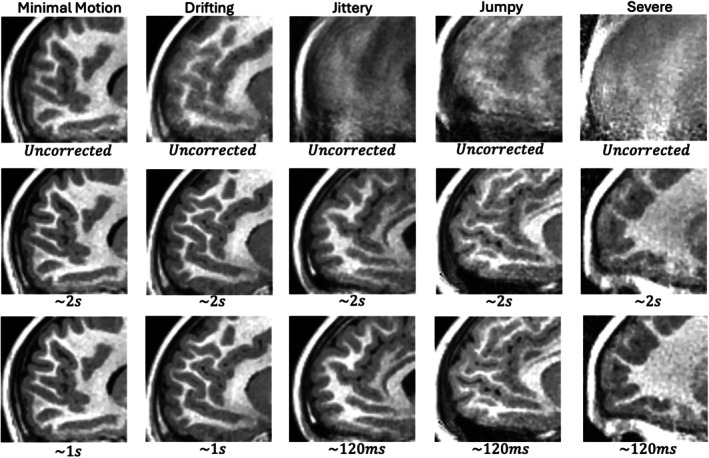
Visual comparison of $T_{1}$‐weighted images reconstructed without and with k‐space motion correction. Each column contains images from one of the five test cases. The top row contains uncorrected images. The middle row contains images corrected at 2s resolution and the bottom row contains images at finest timing resolution used by the algorithm. The corrected images were also reconstructed using the error‐based weighting scheme.

Figure 4 shows uncorrected and corrected images from these experiments in an anterior sagittal view that is particularly sensitive to motion. Whole FOV versions of these images in Figures S1–S5 of the supplement.

When comparing the uncorrected and $N_{s}=1$ (∼2s correction) rows of Figure 4, one finds that motion correction provides a significant benefit in all five cases, with the last three cases being unusable if left uncorrected.

For the minimal motion and drifting cases, increasing the timing resolution from $N_{s}=1$ to $N_{s}=2$ provides a minimal increase in TM, triggering the stopping condition.

For the other three cases, increasing the timing resolution leads to more significant TM increases, with each running up to $N_{s}=16$ segments per block. This is the predetermined finest timing resolution for the experiment.

**FIGURE 5:**
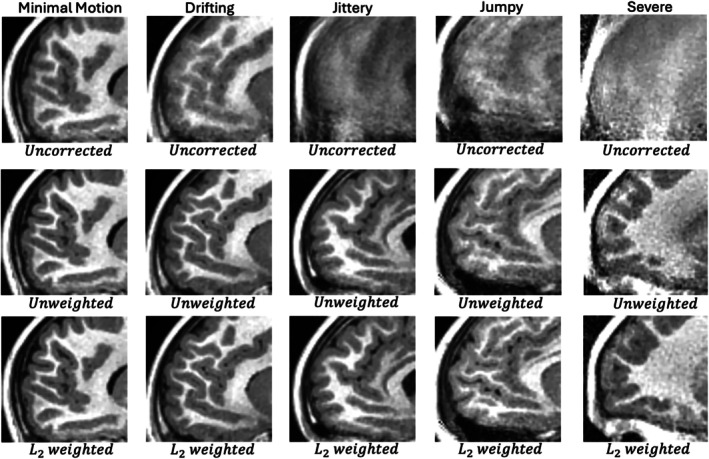
Visual comparison of $T_{1}$‐weighted images reconstructed without motion correction and with motion correction, both with and without $L_{2}$ weighting Equation (6). The top row contains uncorrected images. The middle row contains images corrected without any weighting. The bottom contains images generated with the error‐based weighting scheme, which suppresses higher error data.

**FIGURE 6:**
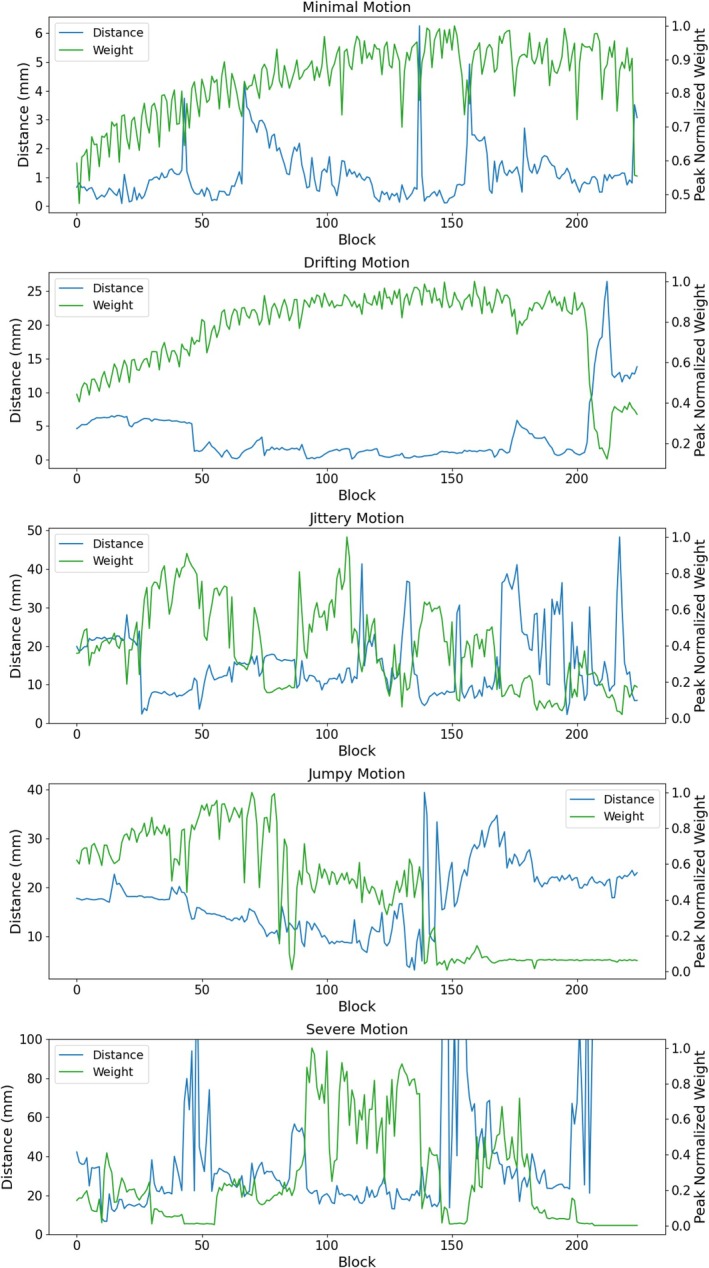
Plots of motion and error weighting for five cases with different motion characteristics. Weights shown here are computed using Equation (6). The distances describe movement of the center voxel of the images shown in Figure 4.

Figure 5 shows images generated with and without the weighting factor G shown in Equations (5) and (6). One may find the full FOV version of these images in Figures S6–S10 of the supplement. All corrected images shown in this figure use the finest timing chosen by the algorithm, which are the same as those given in the bottom row of Figure 4. In this figure, one can see that the weighting scheme provides minimal improvement for the drifting and minimal motion cases. This is due to the fact that these cases are corrected quite well across all blocks, providing weights that are relatively uniform. This is illustrated in Figure 6, which shows plots of weights and motion for the five test cases. The “distances” shown in this plot are tracking motion at the center of the images shown in Figure 5. In Figure 5, it can also be seen that the weighting scheme provides more noticeable improvements in the jittery, jumpy, and severe cases. These particular cases have much more variation in error across blocks. In the jumpy case, it can be seen that weights are quite low for later blocks, despite the distance curve being relatively flat there. This is due to a prescription that has the shoulders in frame for blocks <150, but out of frame during the later blocks. This increases the k‐space error and degrades the quality of motion estimates for those later blocks.

### 3.3 Cortical Thickness Test‐Retest Results

**FIGURE 7:**
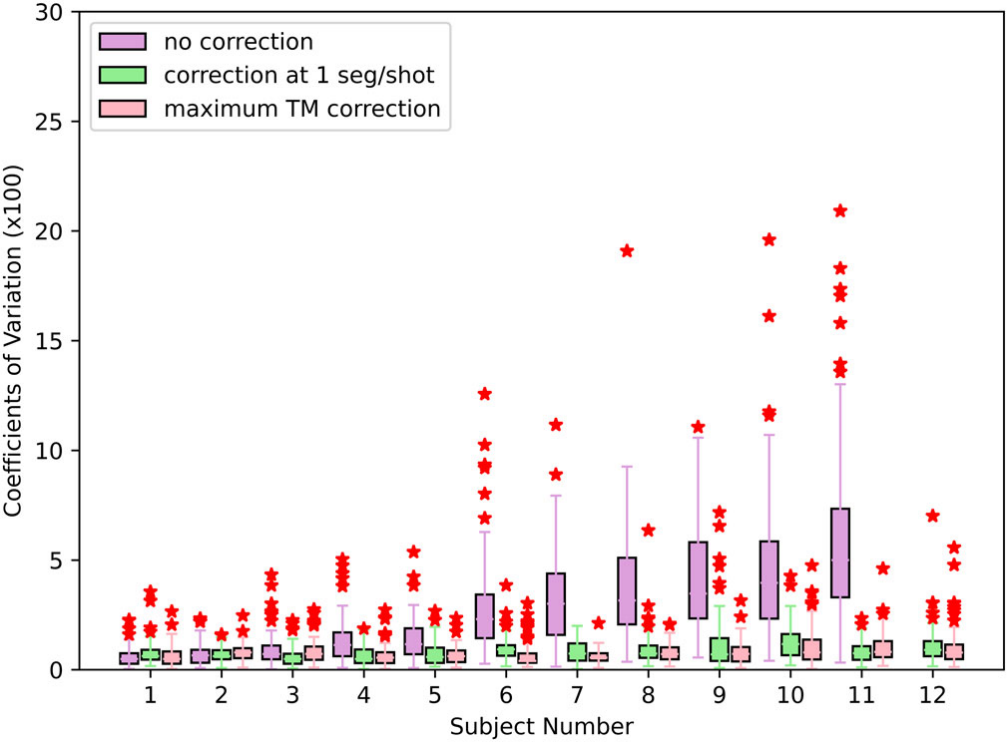
A box plot summarizing the coefficients of variation for cortical thickness estimates for each subject. The pink boxes show the COV obtained when using the highest TM images available for each subject and time point. The subjects are ordered by median uncorrected COV. For subject 12, Freesurfer was unable to process all of the uncorrected images.

A summary of the test‐retest results is presented in Figure 7. This figure shows that the motion correction provides a reduction in the COV for cases with larger amounts of motion. For cases with very little motion, low variation is achieved regardless of the approach.

The mean COV across all subjects for the uncorrected images is 2.73%±1.75%, with Freesurfer unable to process the uncorrected images for one of the subjects due to extreme motion‐induced artifacts. However, Freesurfer was able to process all of the motion‐corrected images. We show the images that could not be processed by Freesurfer, along with its corrected version, in Figure S11–S13 of the supplement. The value for $N_{s}=1$ corrected images is 0.88%±0.21%. The value for the maximum TM images is 0.79%±0.16%. A two‐tailed Wilcoxon test is performed to compare the $N_{s}=1$ and maximum TM results, obtaining a *p*‐value of 0.2. Thus, the improvement seen in this case is not statistically significant. The same test provides a *p*‐value of 0.003 when comparing uncorrected and $N_{s}=1$ results.

## 4 Discussion

This paper describes the application of a k‐space‐based motion‐correction method to 3D T1‐weighted radial imaging data capable of correcting for motions as rapid as about 100ms. For this method, an iterative and automated procedure based on image sharpness was developed to choose the rate of correction that results in the sharpest images. Moreover, an error‐based weighting scheme was introduced to suppress poorly corrected RF‐blocks during image reconstruction.

In Section 3.2, the effectiveness of this motion‐correction technique was demonstrated on cases with distinct motion characteristics, obtaining qualitative improvements in all cases. For the three most severe cases, it was shown that increased timing resolution and error‐based weighting provided visually noticeable improvements in image quality. For the two less severe cases, the corrected images looked similar regardless of timing resolution or weighting.

Section 3.3 showed the effect that this motion‐correction technique has on estimates of cortical thickness. On average, it was found that motion correction significantly lowered the coefficients of variation. The effect of timing resolution on these cortical thickness estimates was also investigated, with hints of improvement being found with increased timing resolution. However, this improvement was not statistically significant. It should be noted that the sample size is fairly small.

## 5 Conclusion

In conclusion, we demonstrated that this k‐space based motion‐correction method is effective for 3D $T_{1}$‐weighted radial imaging data, producing visually improved images and more consistent cortical thickness estimates. Moreover, the fine‐scale timing resolution and error‐based weighting possible with this method were shown to provide additional qualitative improvement in images with severe motion effects. These benefits were all achieved by applying the method to actual image data, without any need for additional sensors or navigator data.

## Supporting information




**Data S1**: Supporting Information.
