# Supplementary material for: Self‐Navigated, Retrospective, Data‐Consistent Motion Correction for MPnRAGE
Source: Magn Reson Med. 2025 Oct 13;95(3):1429–39. doi: 10.1002/mrm.70126 (PMC12631670; doi:10.1002/mrm.70126)
Supplement: Supplementary file 1 — Data S1: Supporting Information. [file MRM-95-1429-s001.pdf]

Supplemental Materials for
“Self-Navigated, Retrospective, Data-Consistent Motion Correction for MPnRAGE"

Whole FOV images

- The next 5 slides will show whole FOV slices taken from the images used to make figure 4 of the paper.

Jittery

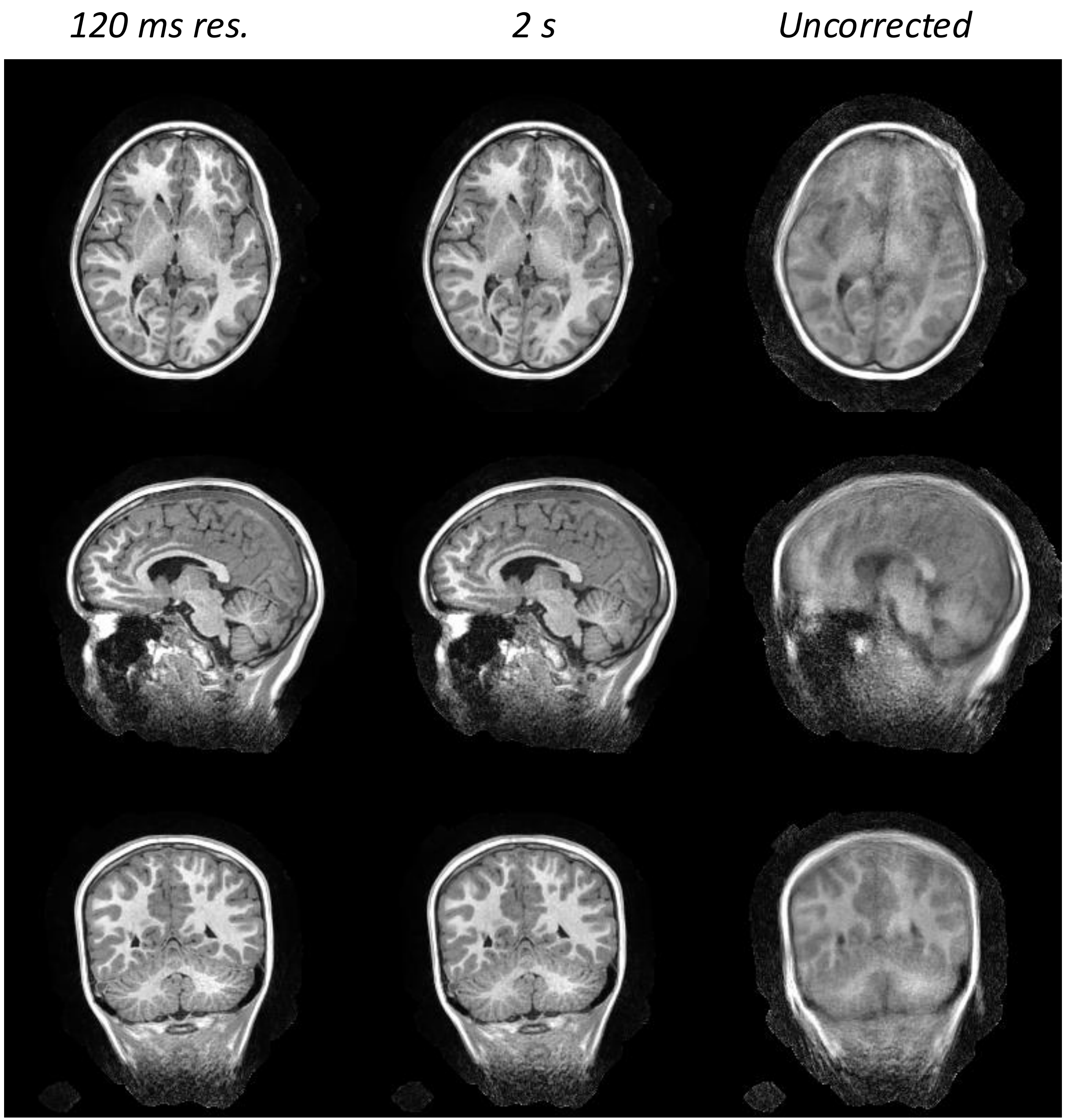

Fig. S1

Jumpy

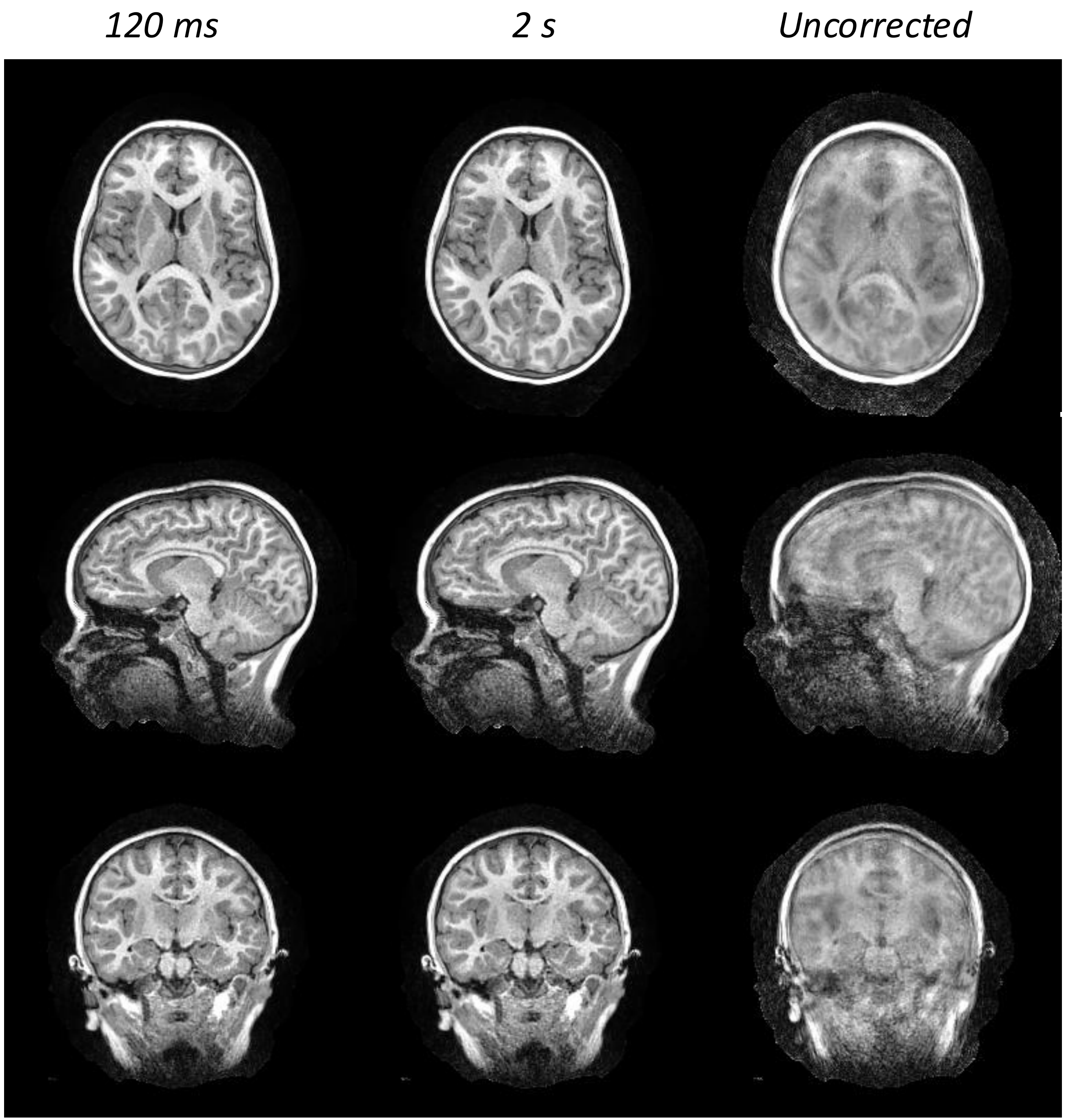

Fig. S2

Drifting *1 s* *2 s* *Uncorrected*

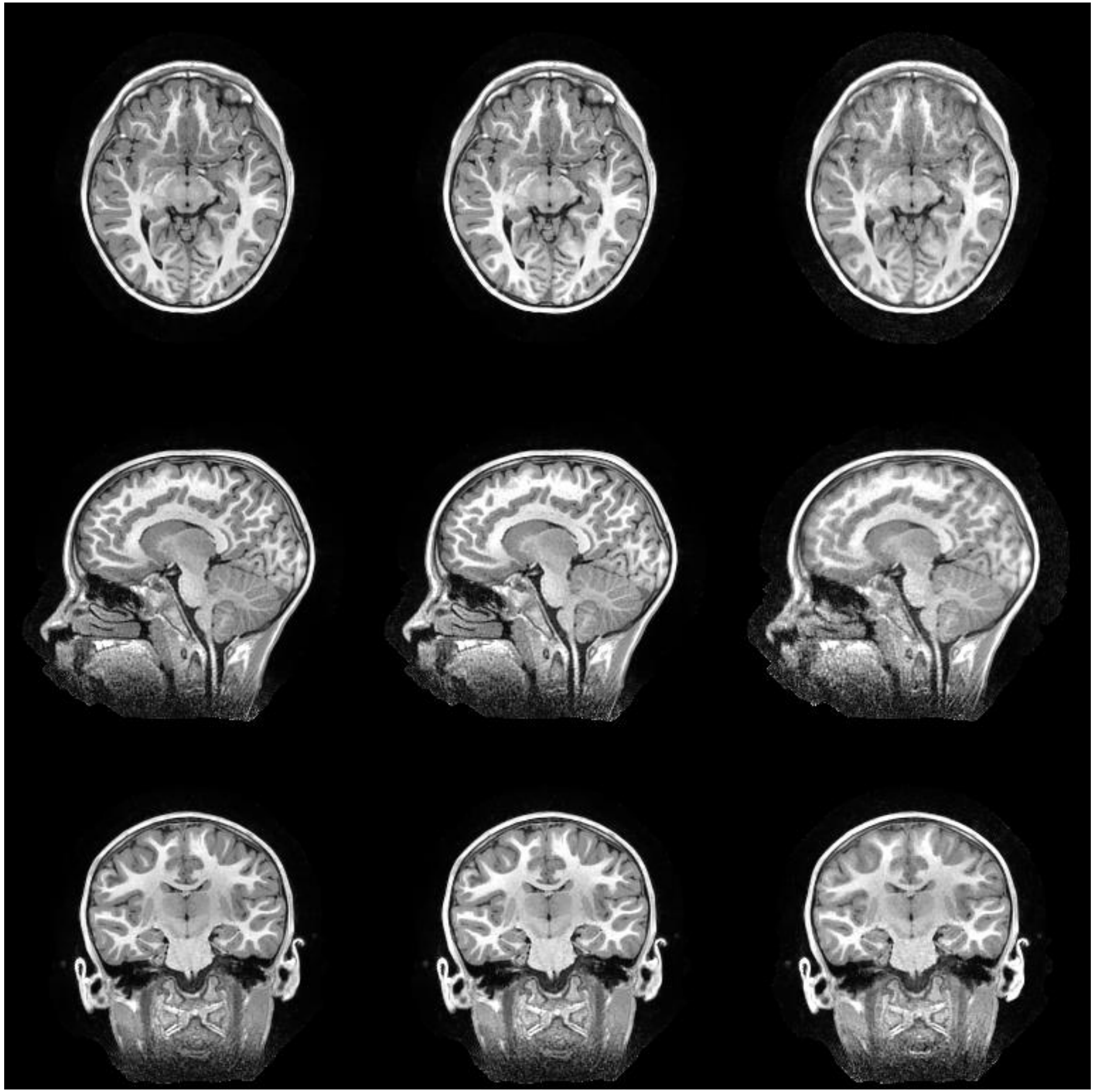

Fig. S3

Minimal motion

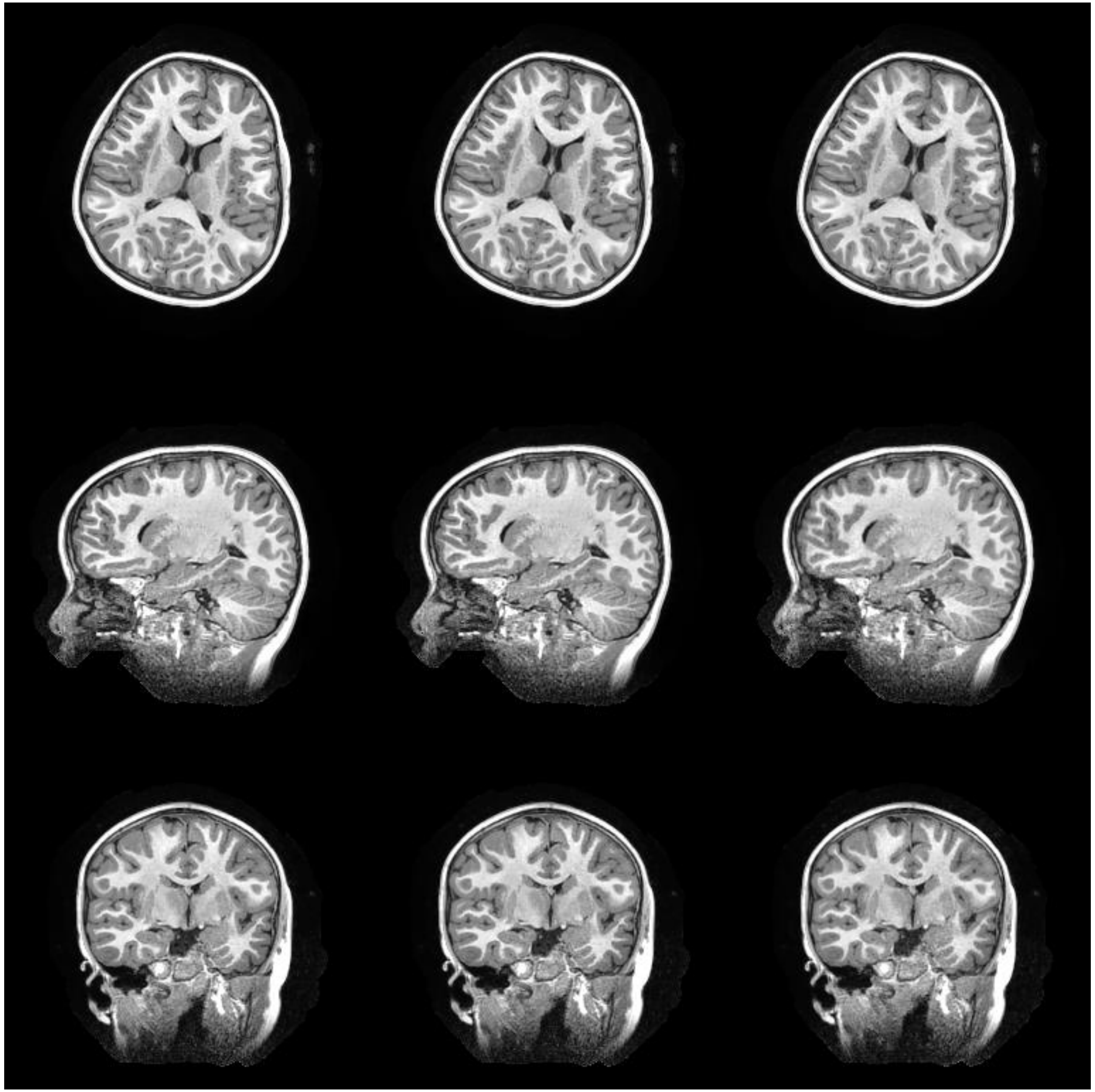

Fig. S4

Severe

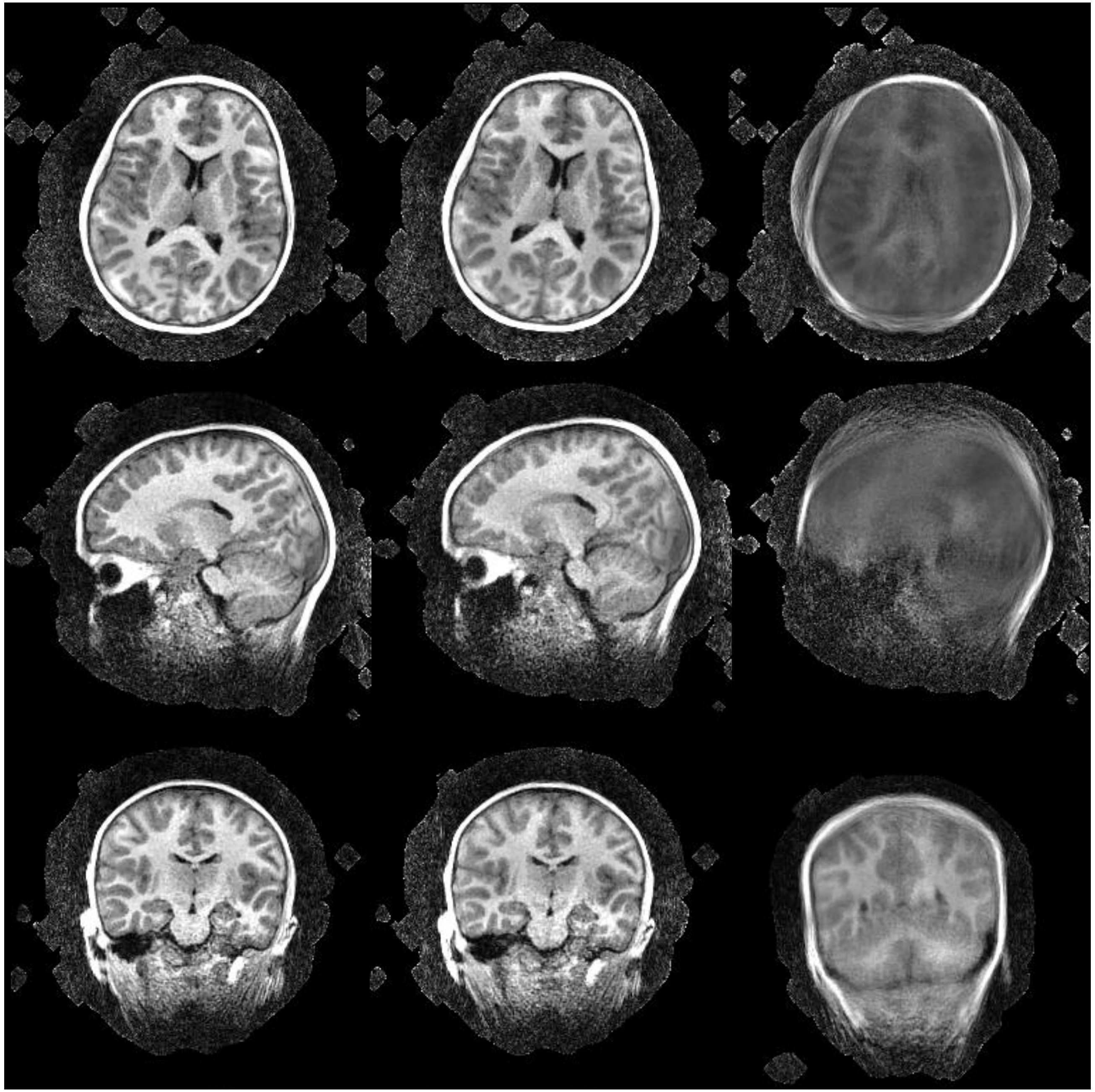

Fig. S5

Whole FOV for weighting comparisons

- The next 5 images show the whole FOV for the weighted and unweighted examples shown in figure 5 of the paper.

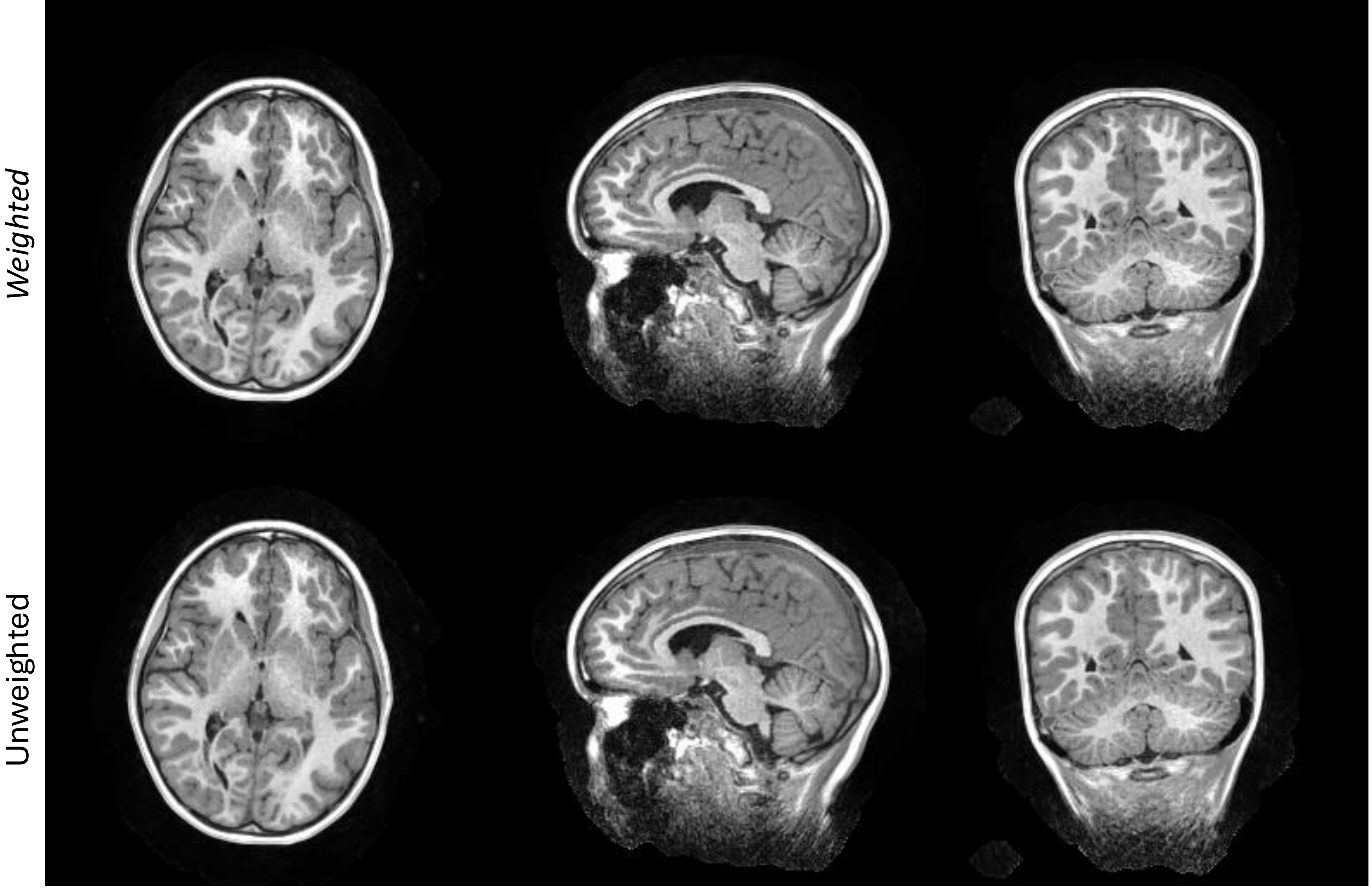

Jittery

Fig. S6

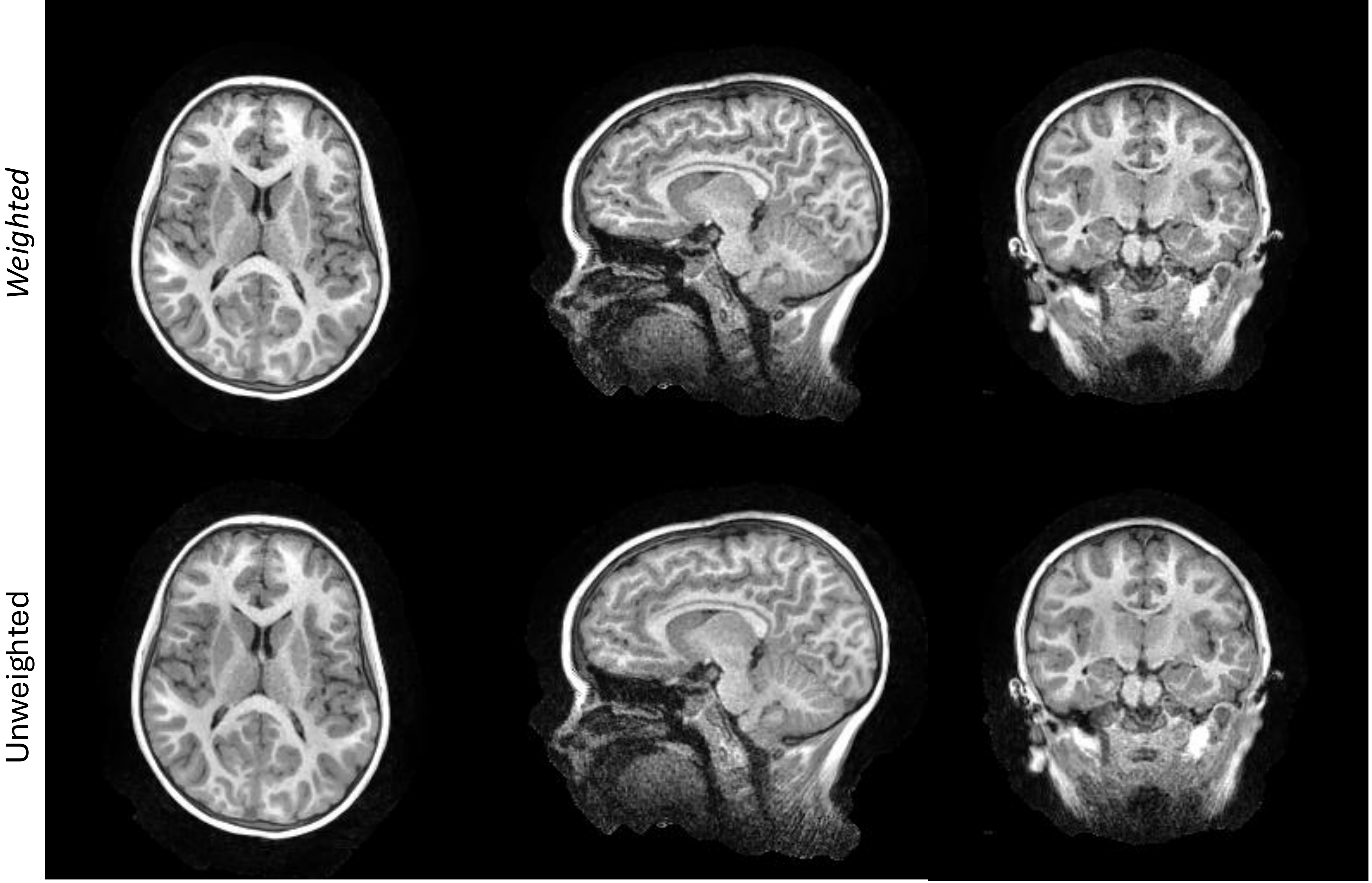

Jumpy

Fig. S7

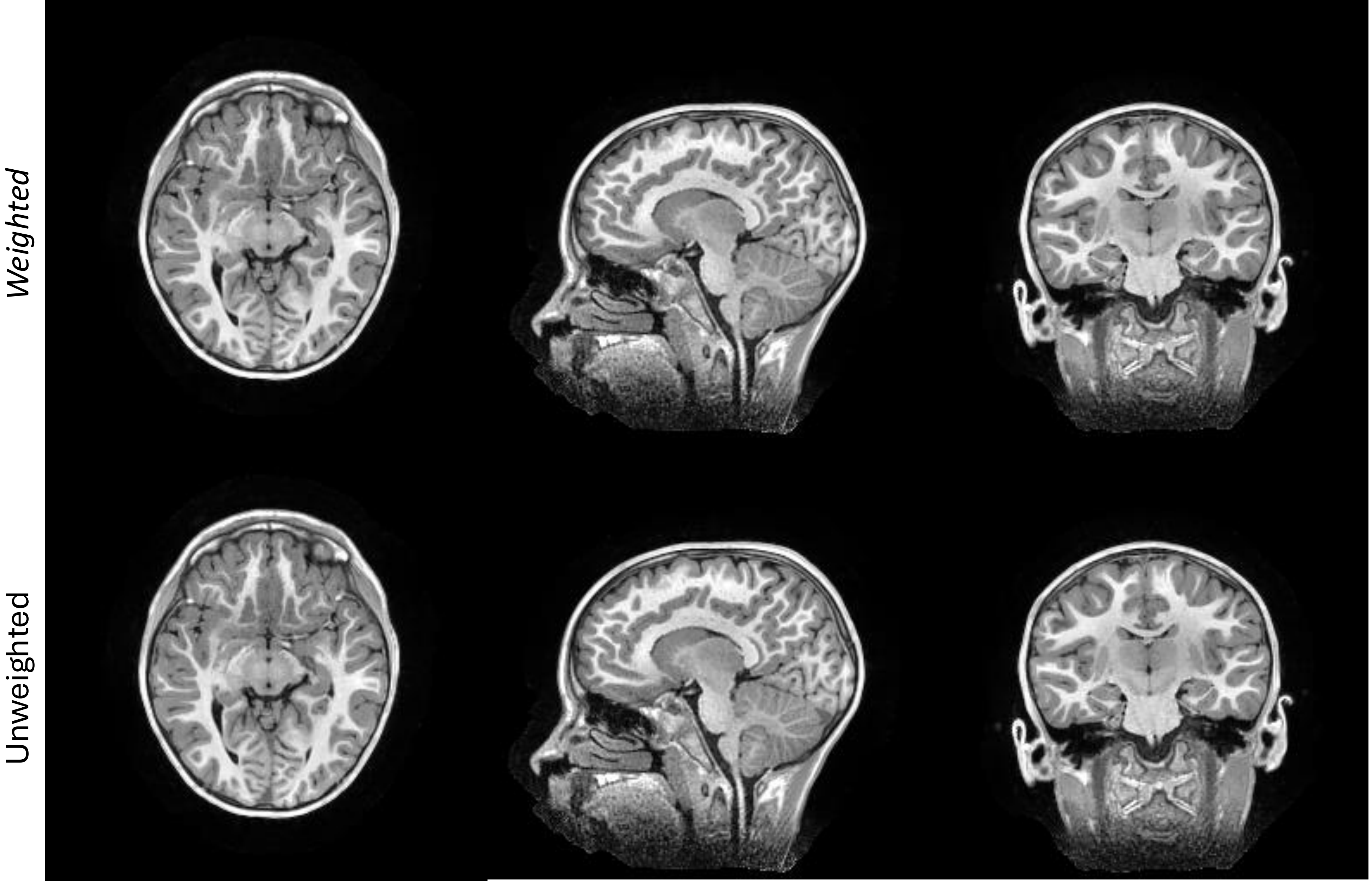

Drifting

Fig. S8

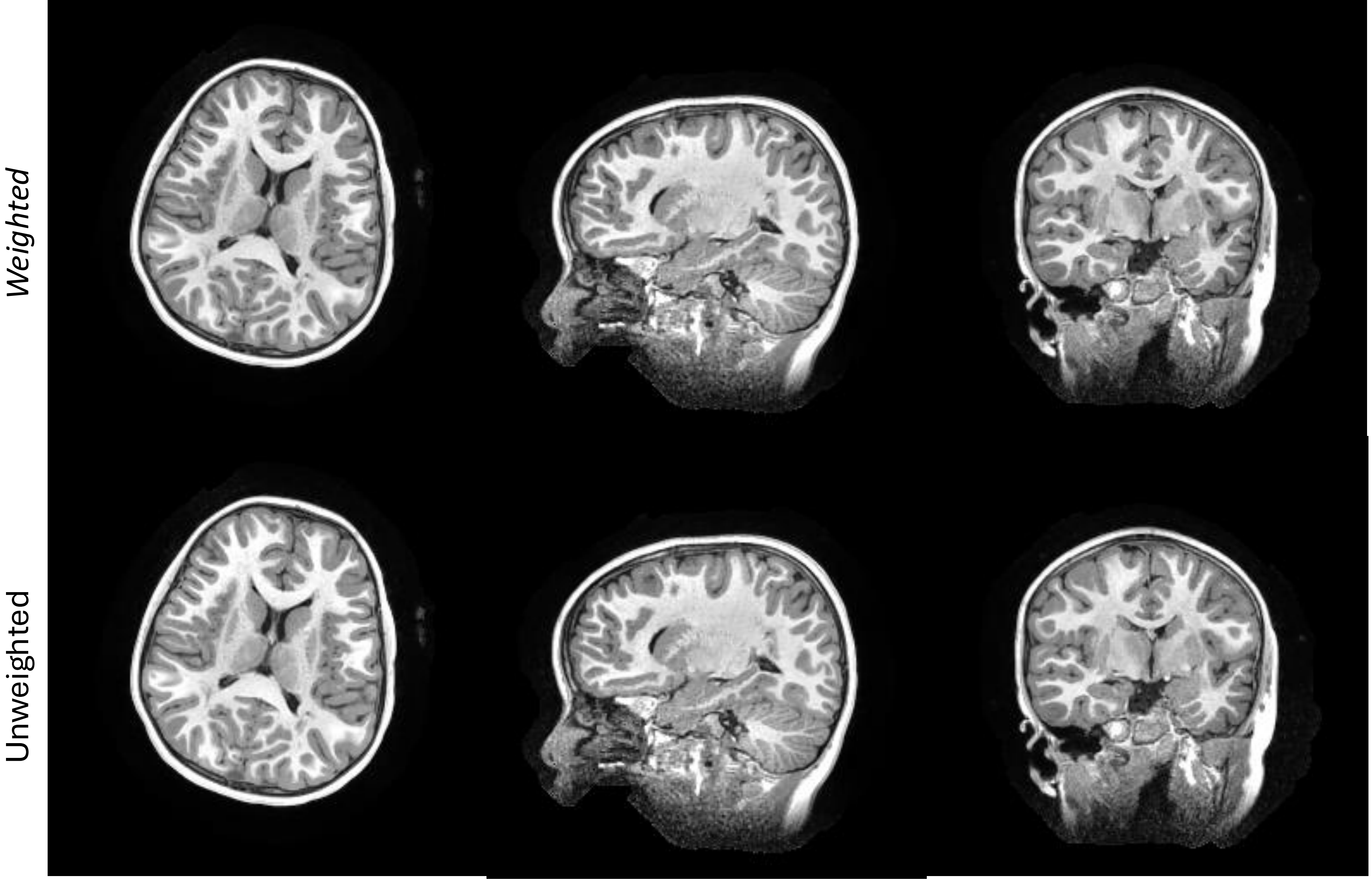

Minimal Motion

Fig. S9

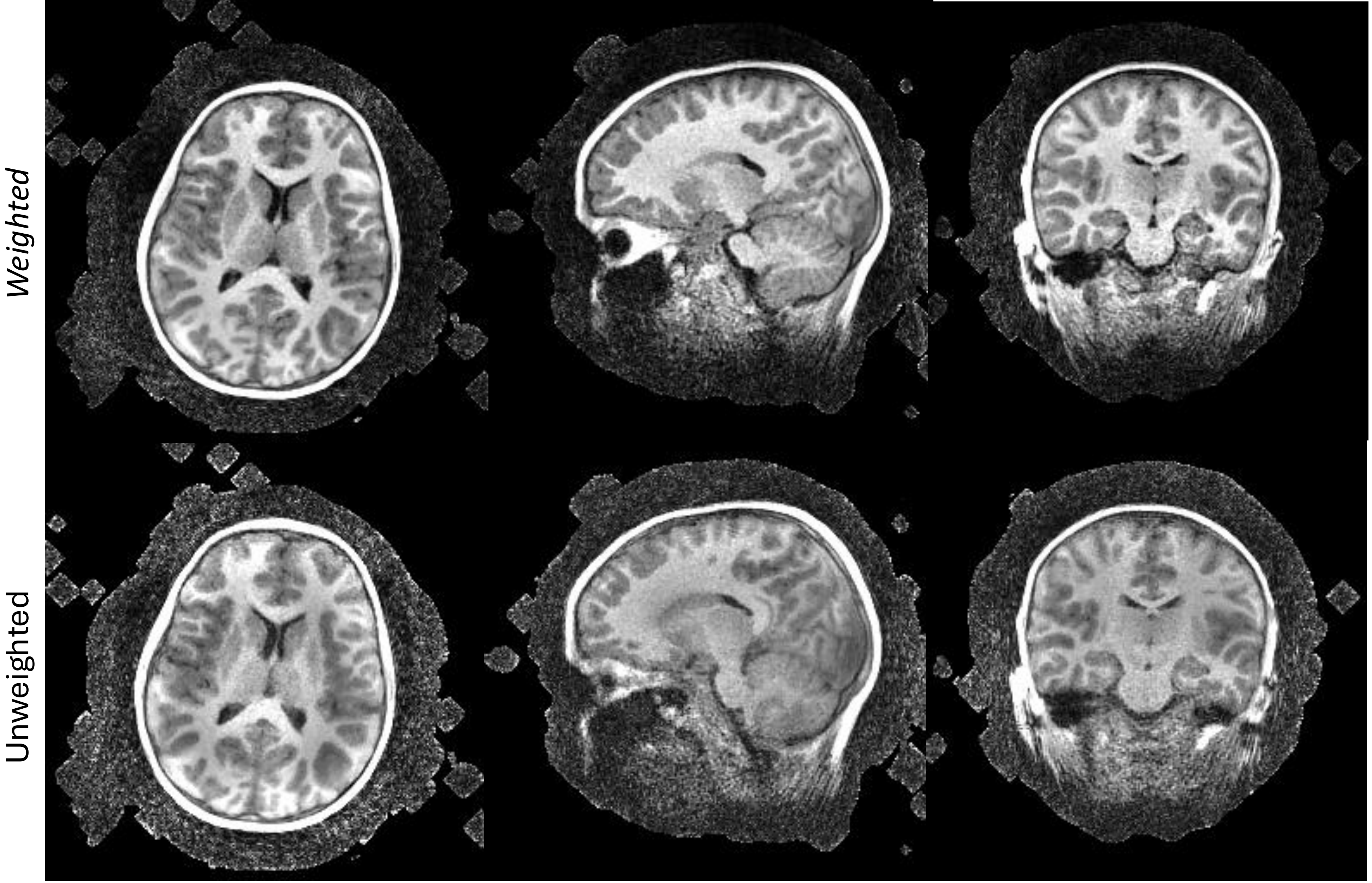

Severe

Fig. S10

Image unable to be processed by FreeSurfer

- When presenting the boxplot in figure 7 of the paper, we mention that FreeSurfer was unable to process one of the uncorrected images.
- In the next 3 slides, we show slices of this unprocessed image along with its motion-corrected counterpart.

The uncorrected image that FreeSurfer was unable to process.

Corrected version

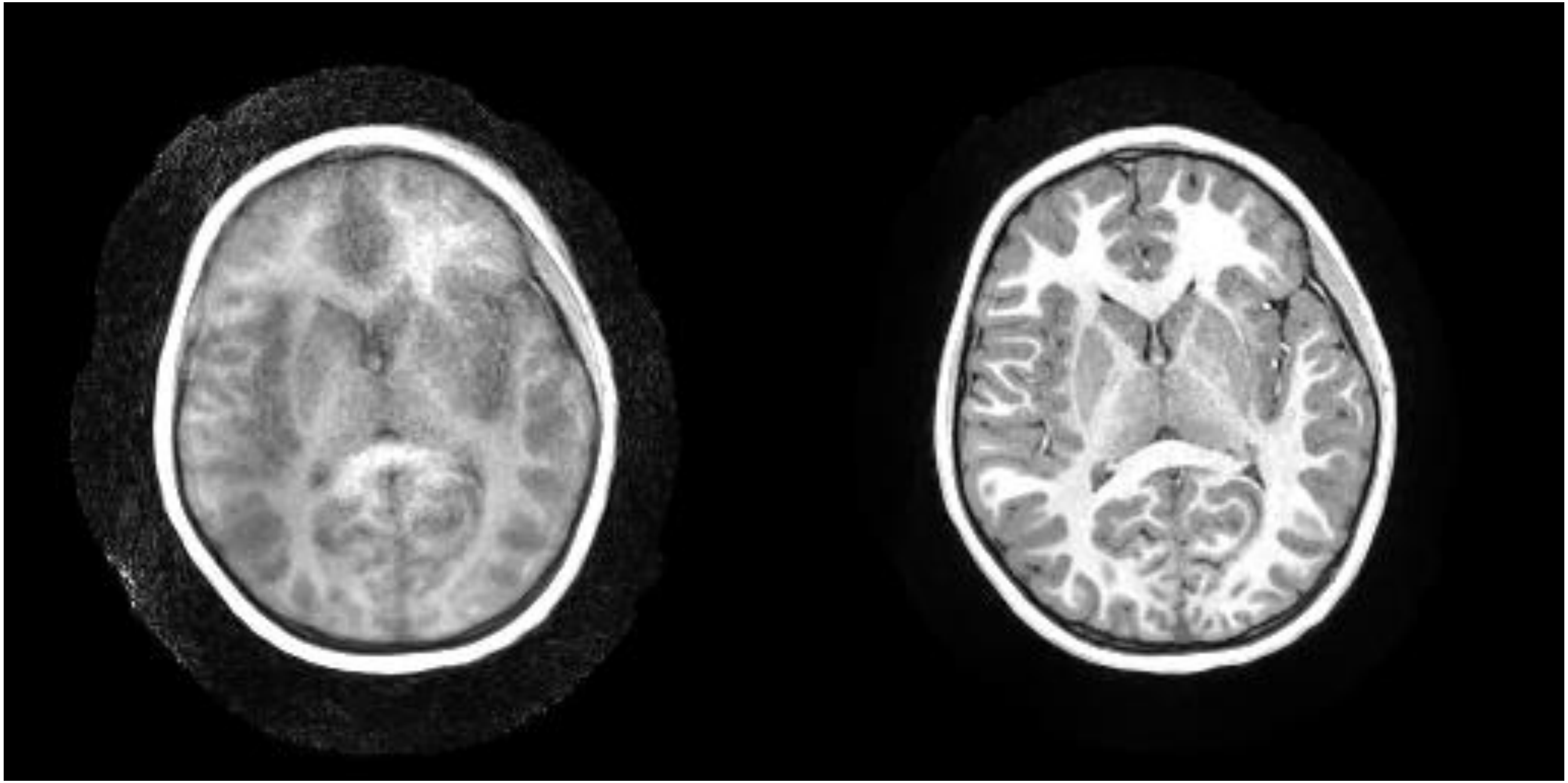

Fig. S11

The uncorrected image that FreeSurfer was unable to process.

Corrected version

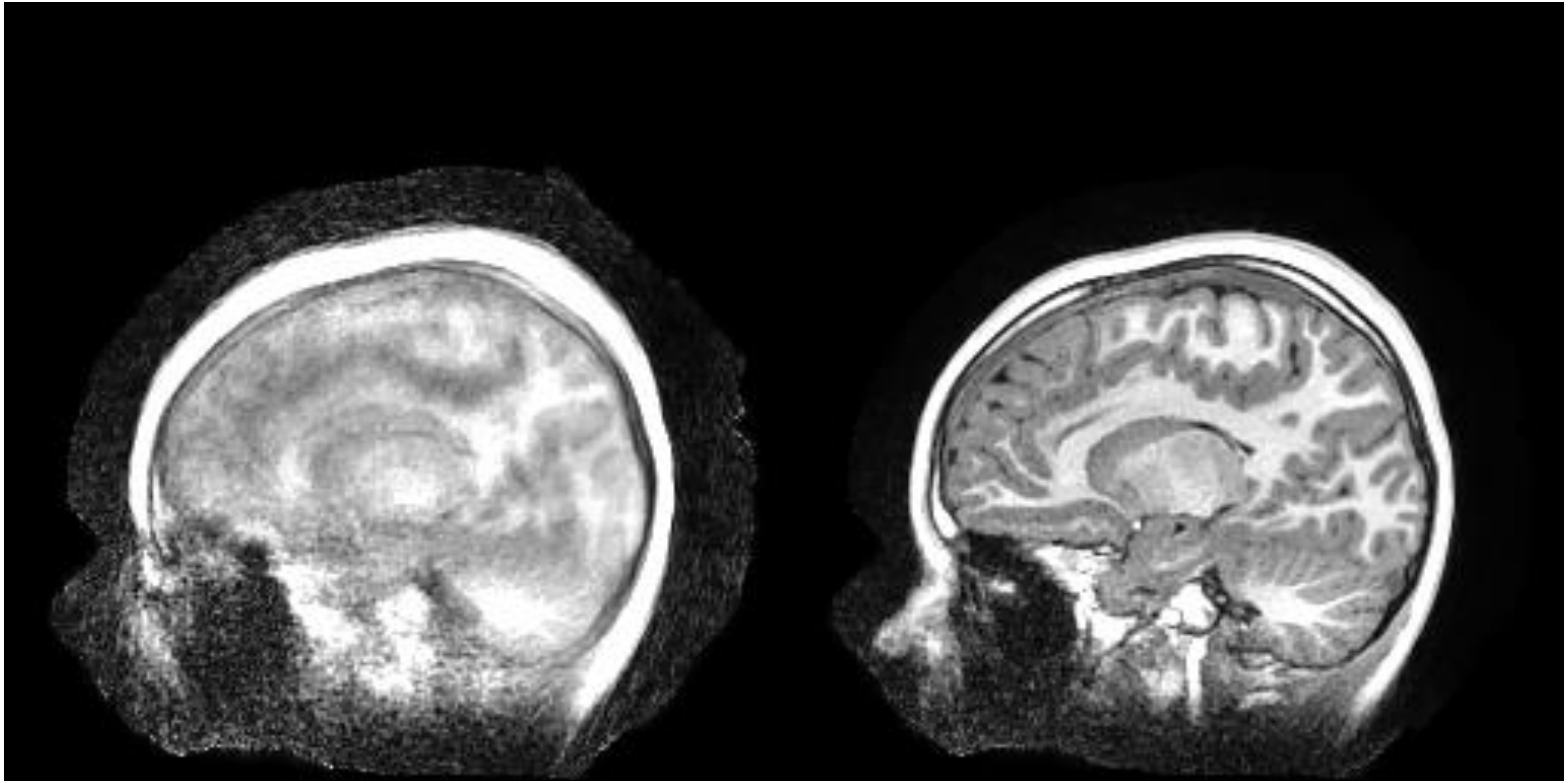

Fig. S12

The uncorrected image that FreeSurfer was unable to process.

Corrected version

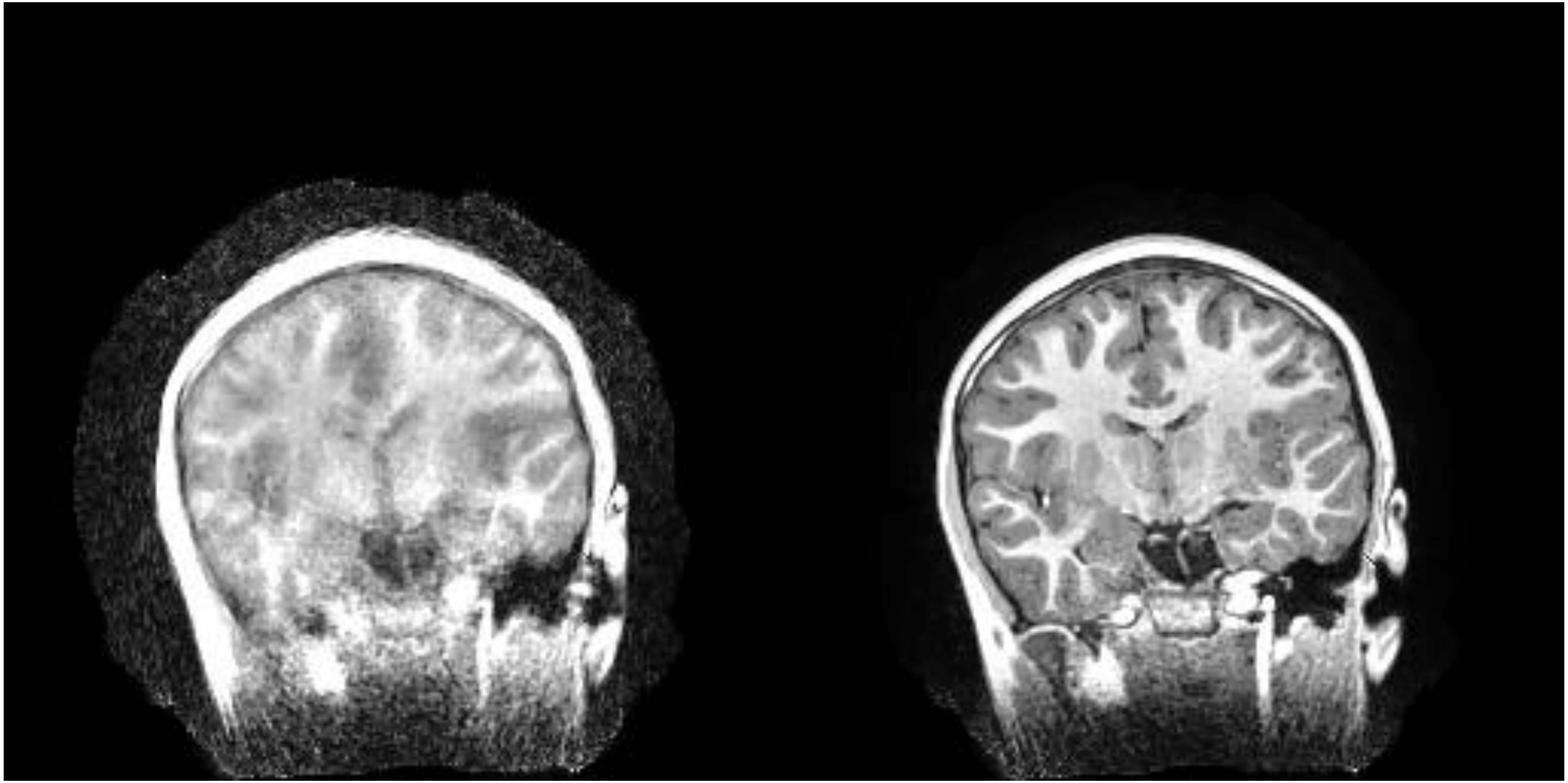

Fig. S13

Simulated Example

Description:

1.) Generated motion parameters randomly.

* Rotations drawn from a normal distribution with a 3 degree standard deviation.

* Translations also drawn randomly, but with a 3 mm standard deviation.

* One set of parameters per block.

2.) Apply these motions to a motion-free image, use this to generate motion-corrupted k-space data.

3.) Apply the method to this data, checking for parameter accuracy and quality of the corrected image.

Simulated Parameters I

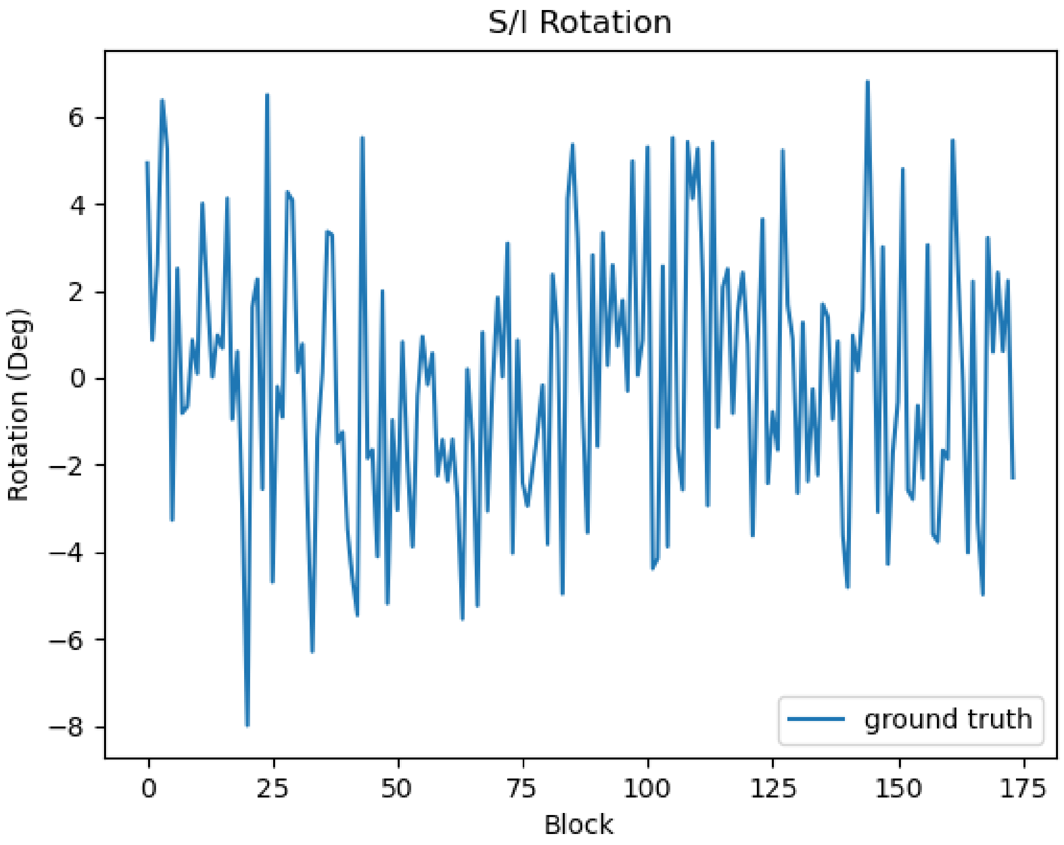

Fig. S14

Simulated Parameters II

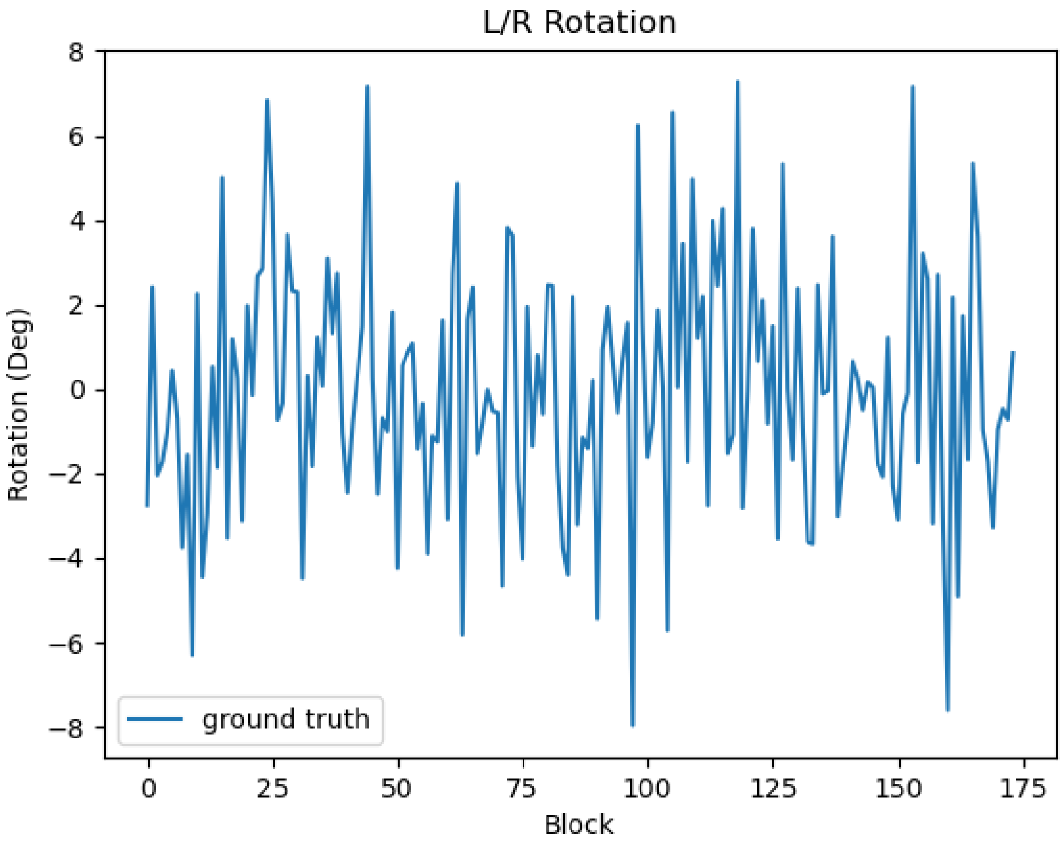

Fig. S15

Simulated Parameters III

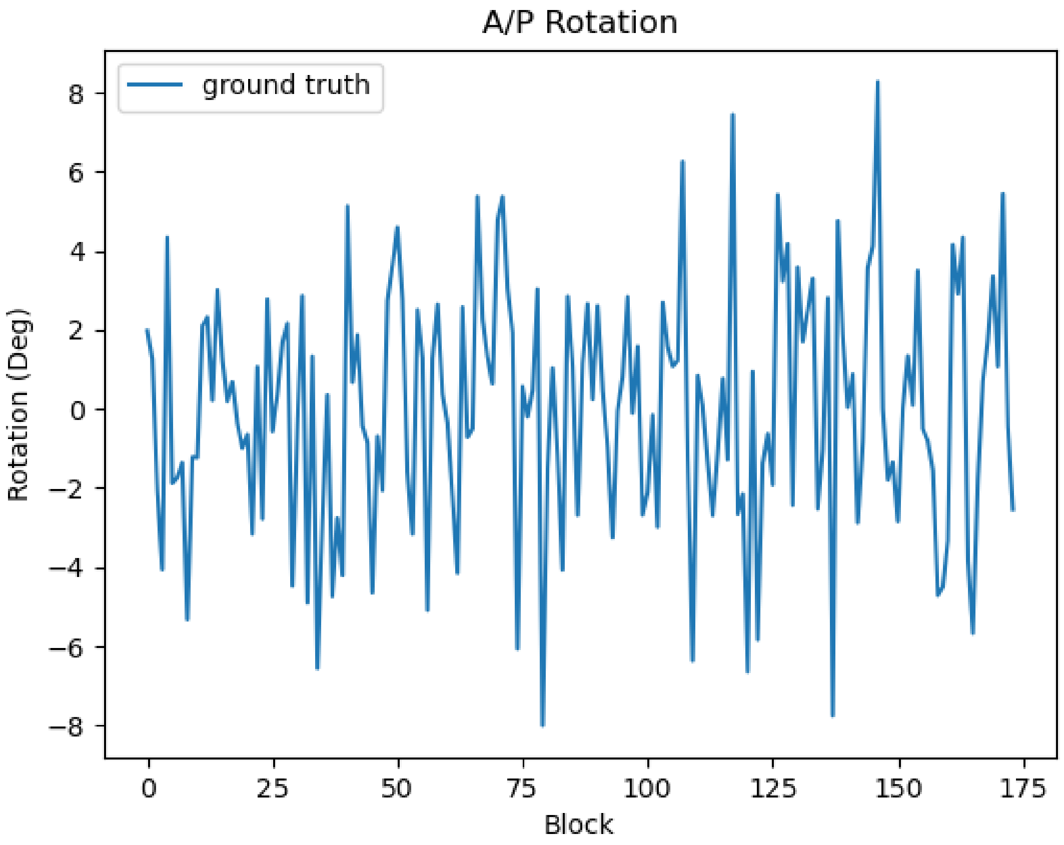

Fig. S16

Simulated Parameters IV

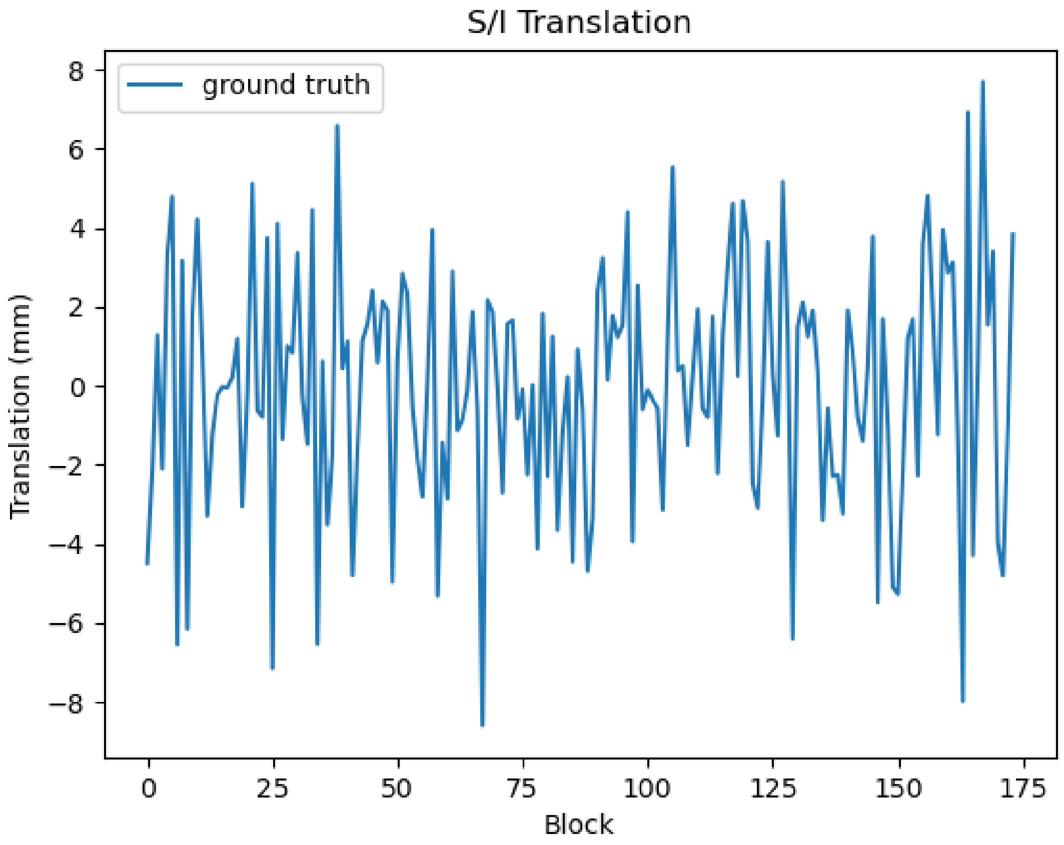

Fig. S17

Simulated Parameters V

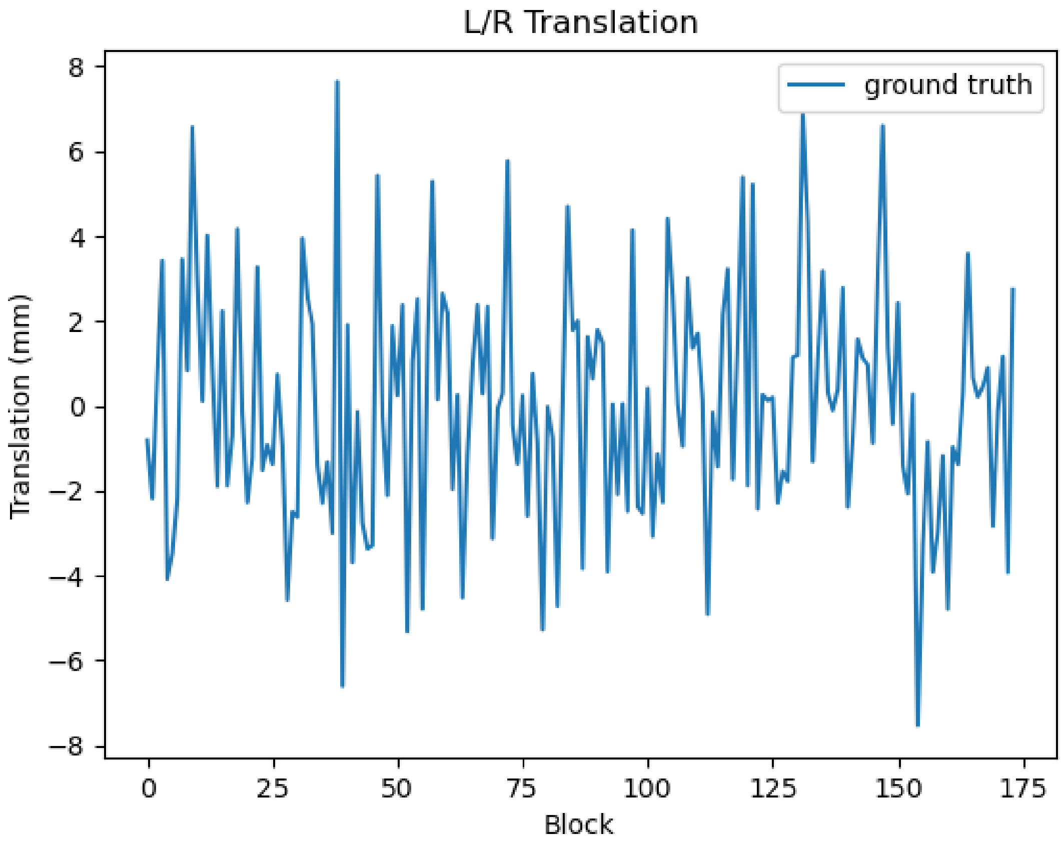

Fig. S18

Simulated Parameters VI

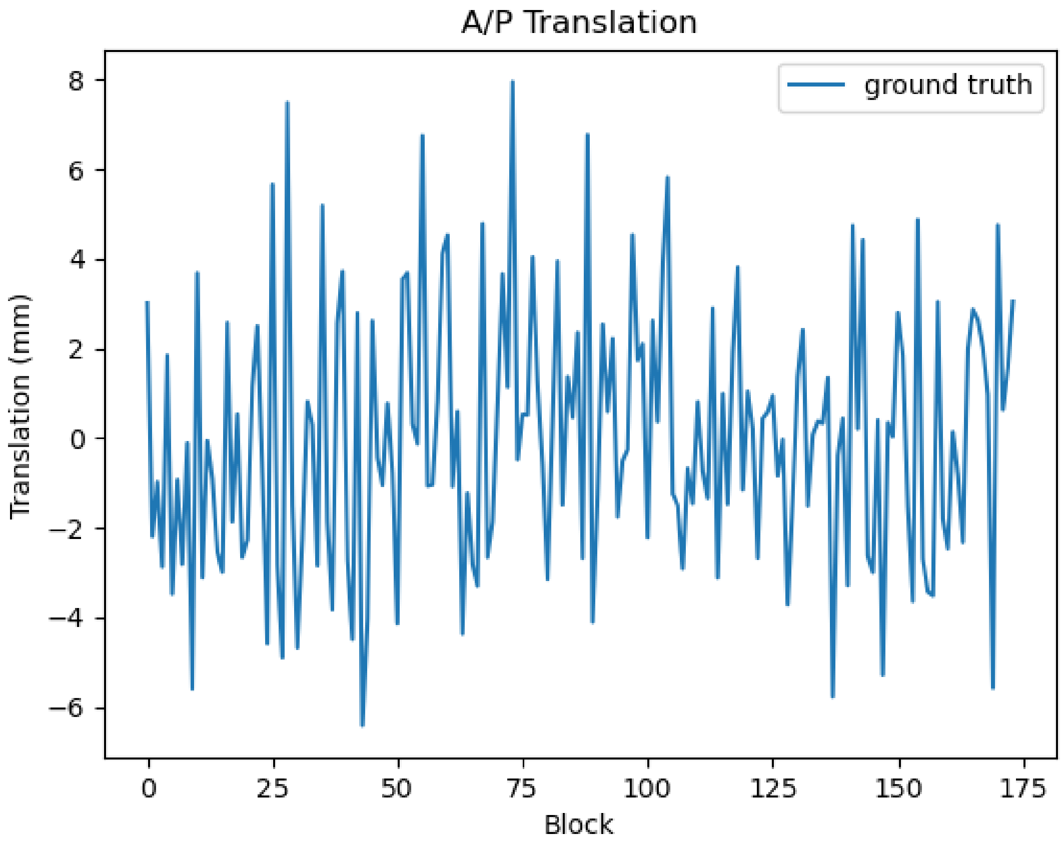

Fig. S19

Original and Motion Applied Images

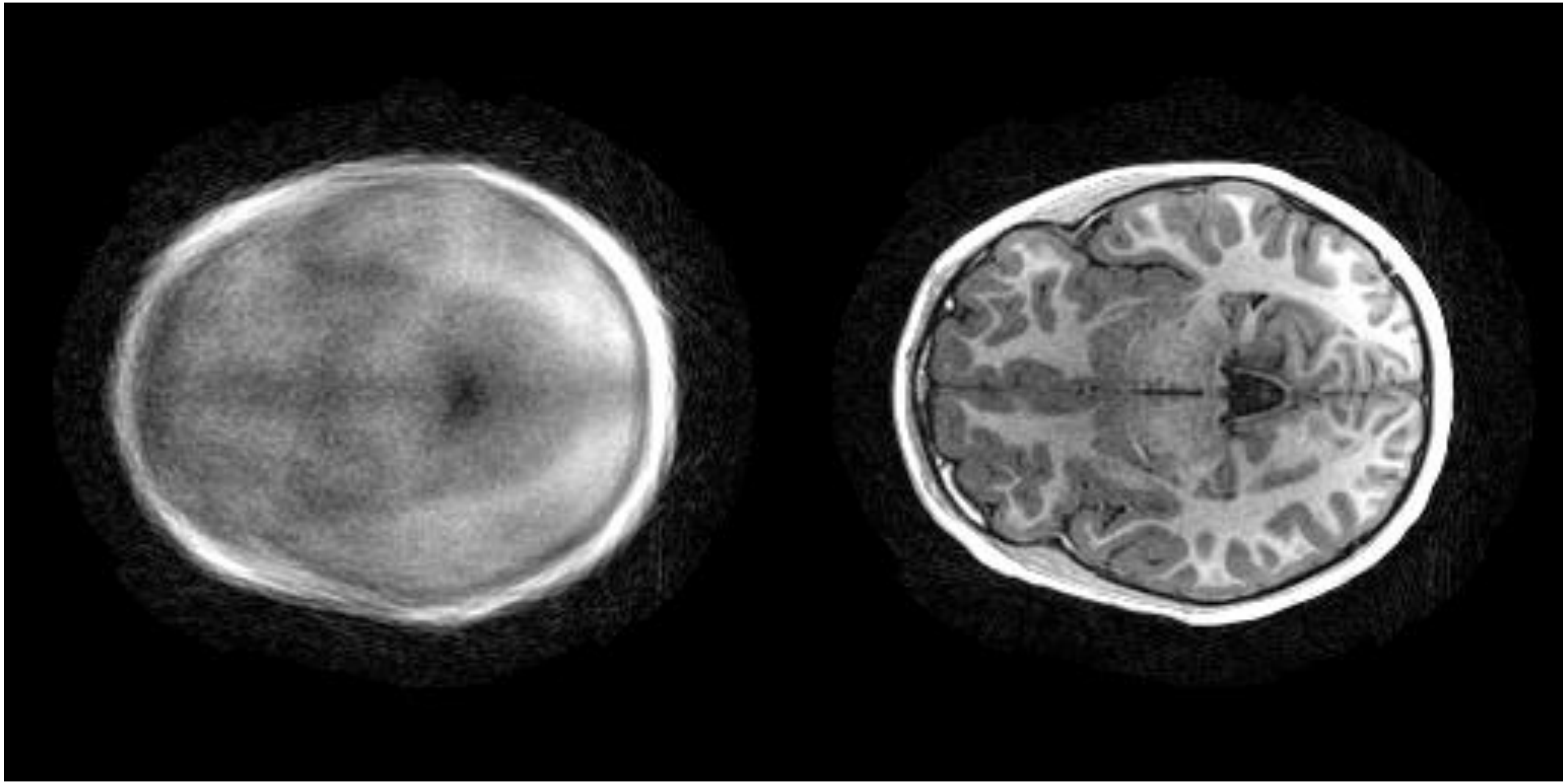

Fig. S20

Estimated Parameters I

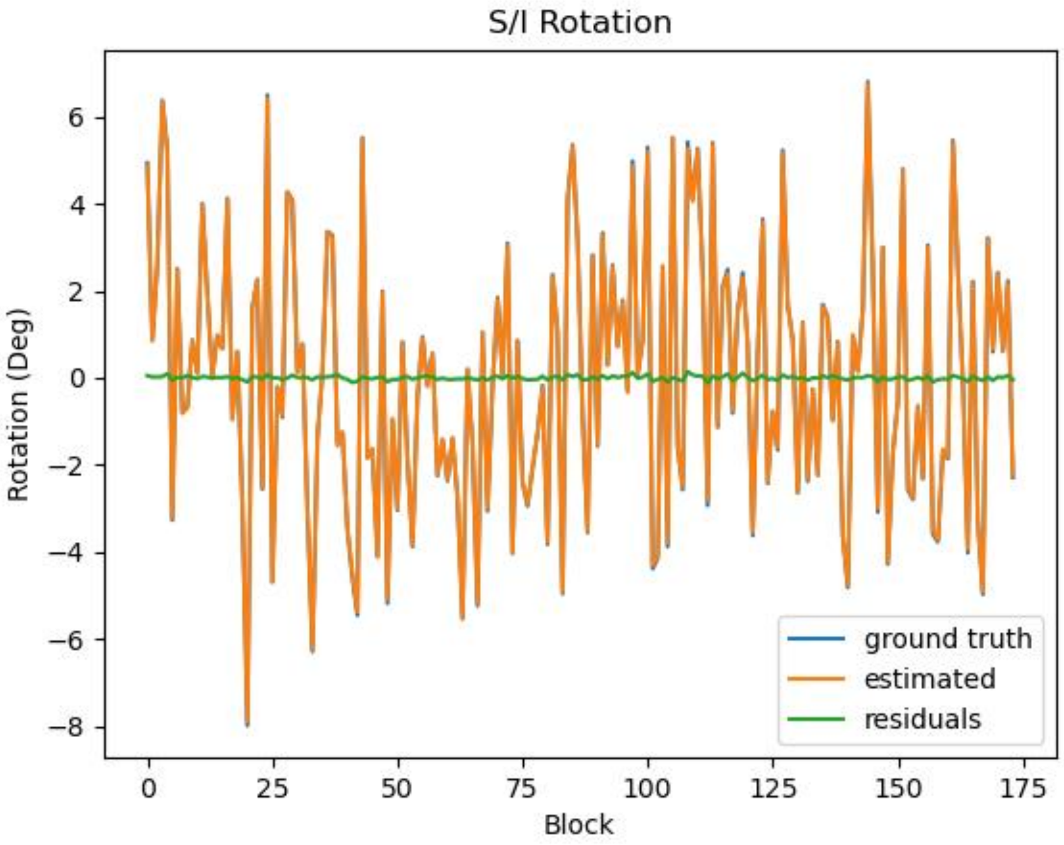

Fig. S21

Estimated Parameters II

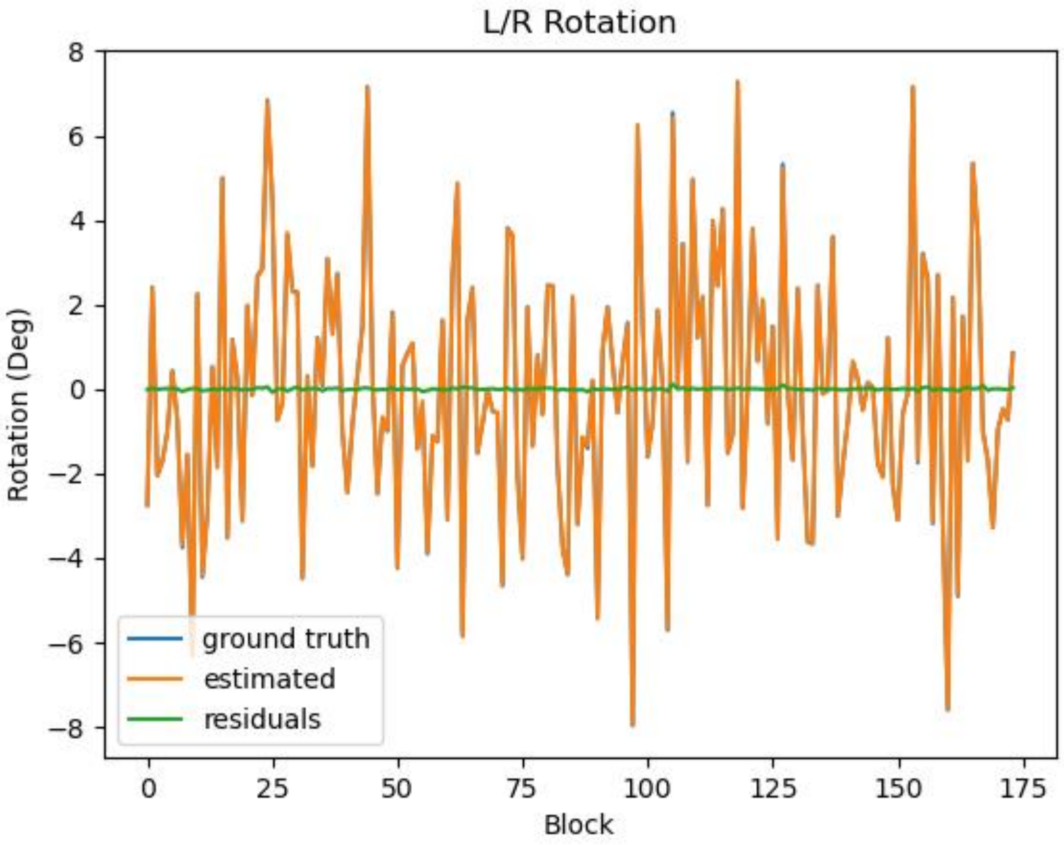

Fig. S22

Estimated Parameters III

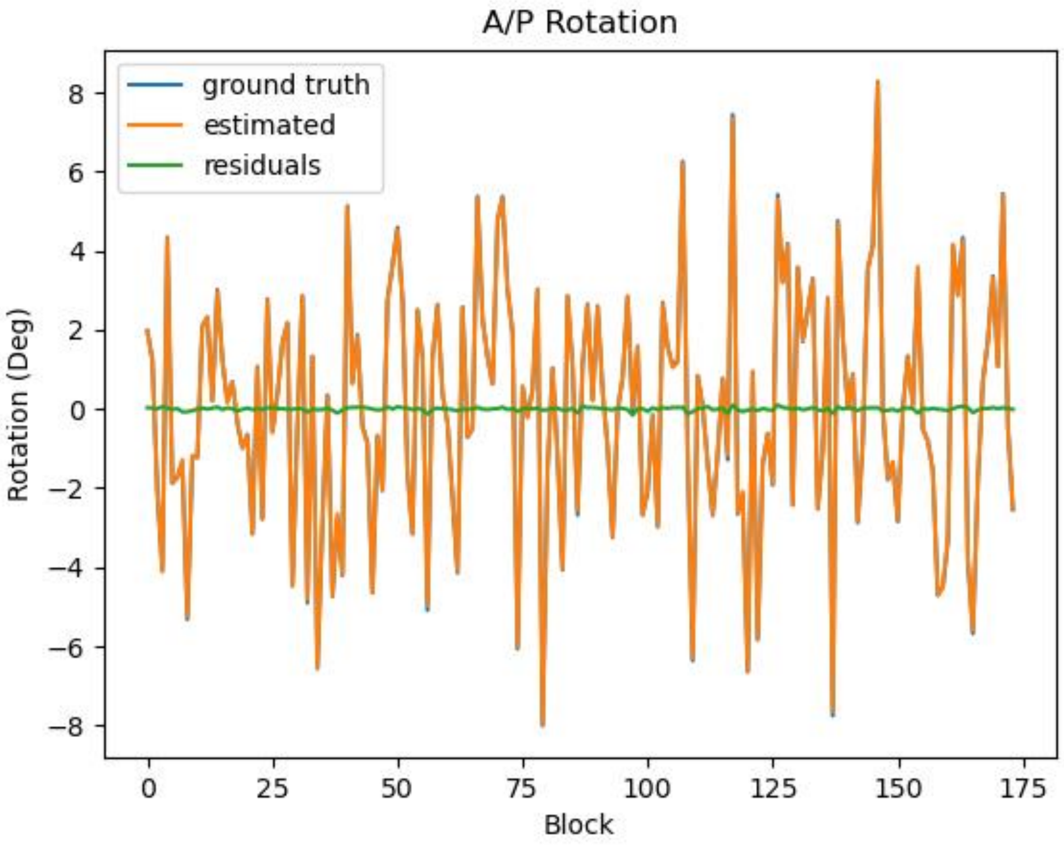

Fig. S23

Estimated Parameters IV

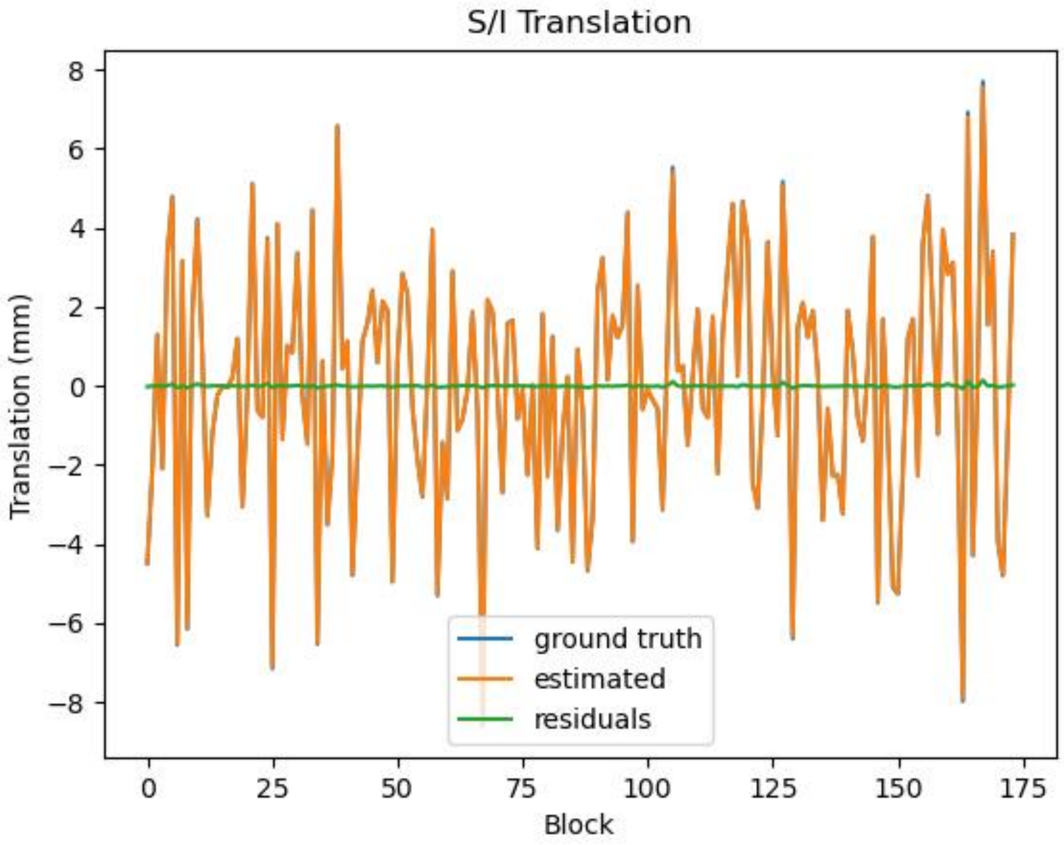

Fig. S24

Estimated Parameters V

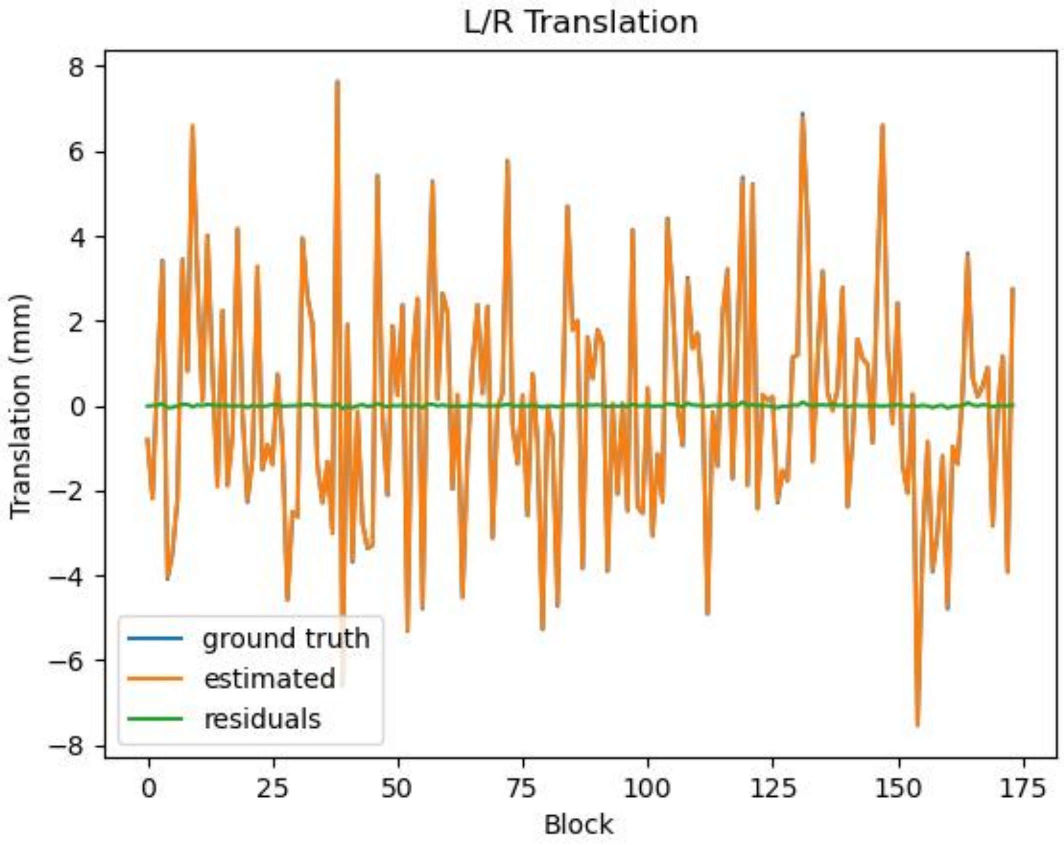

Fig. S25

Estimated Parameters VI

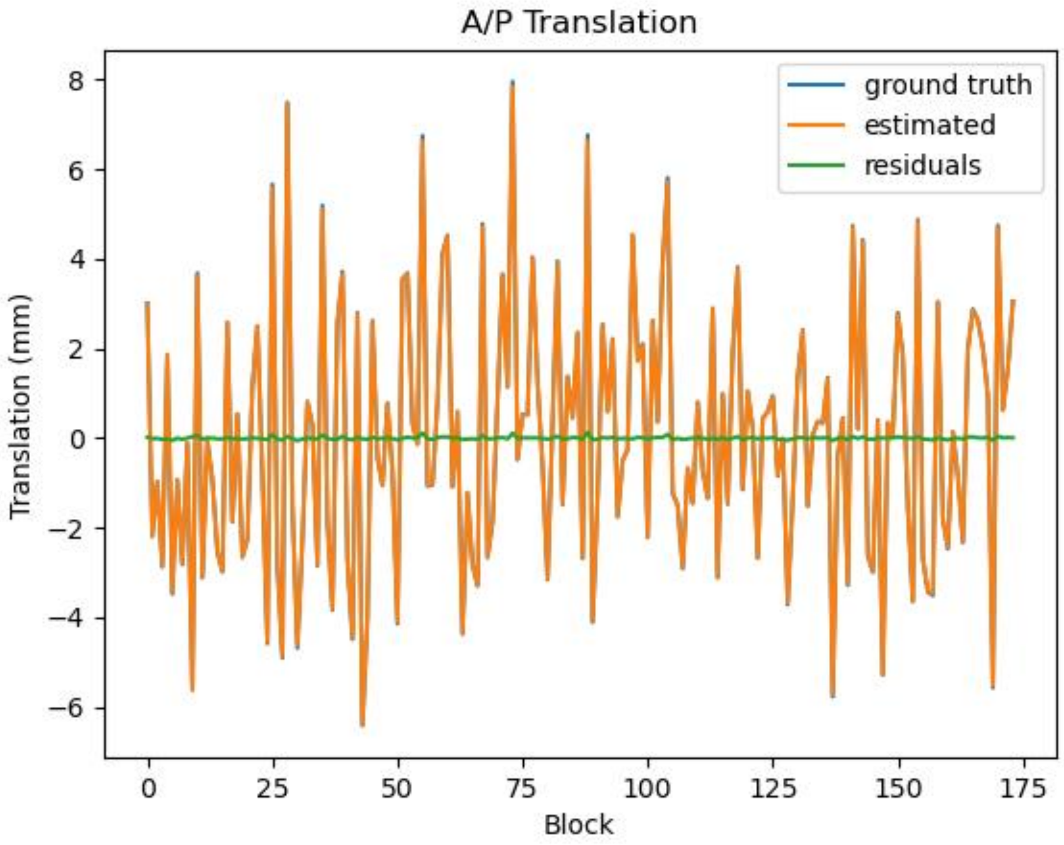

Fig. S26

Results

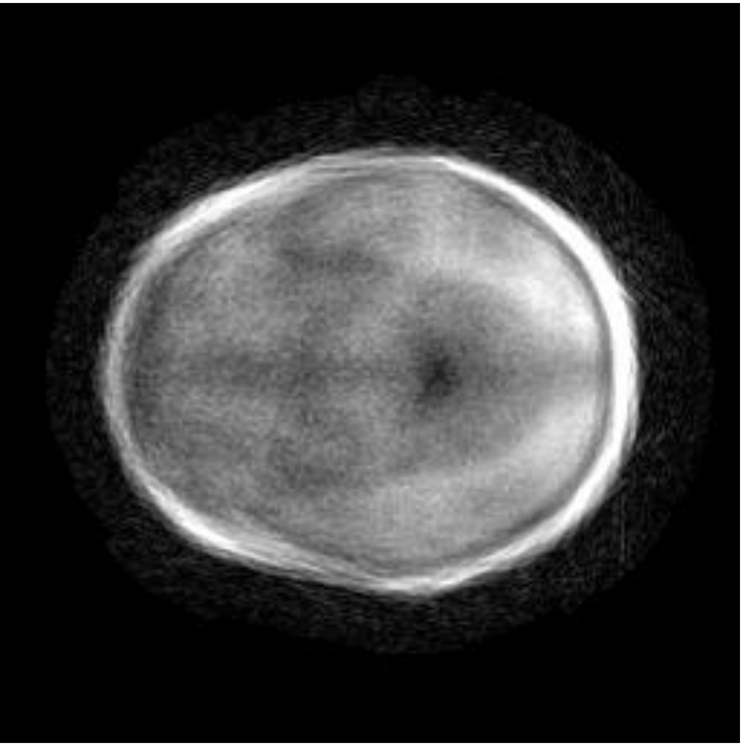

Motion Corrupted

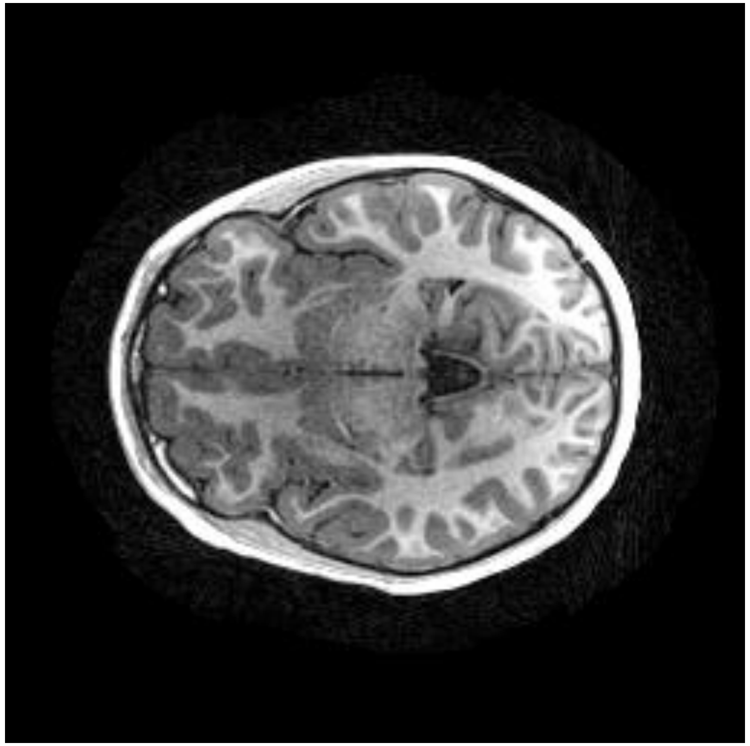

Original

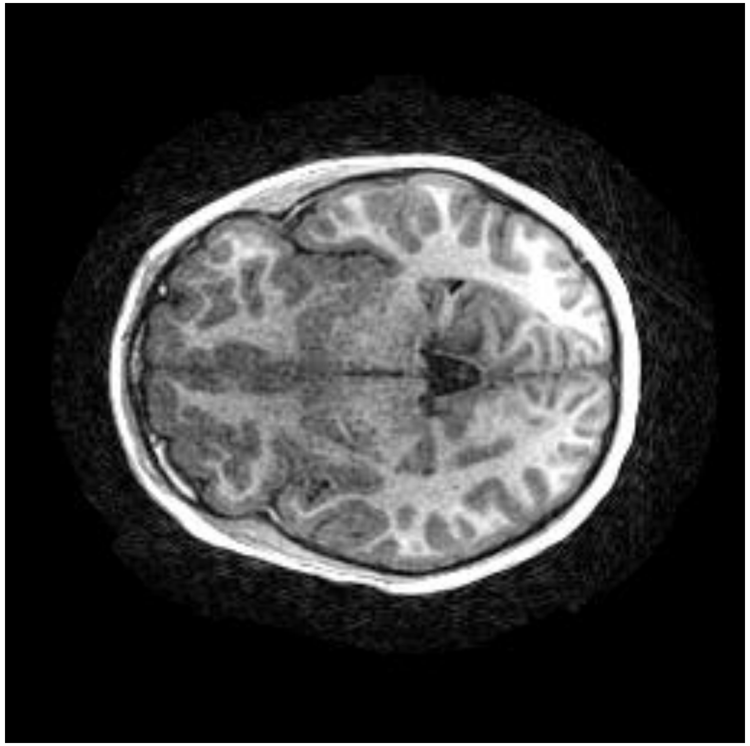

Corrected

Fig. S27

Weighting Tests

- In the paper, we mention testing the L2 norm, L1 norm and the square root of the L2 norm we developing our weighting scheme.
- The following slides show slices from Jumpy, Severe and Jittery cases to show some examples from these norm tests.
- We have left out the drifting and minimal motion cases as the weighting doesn't have much effect in these cases, regardless of norm chosen.

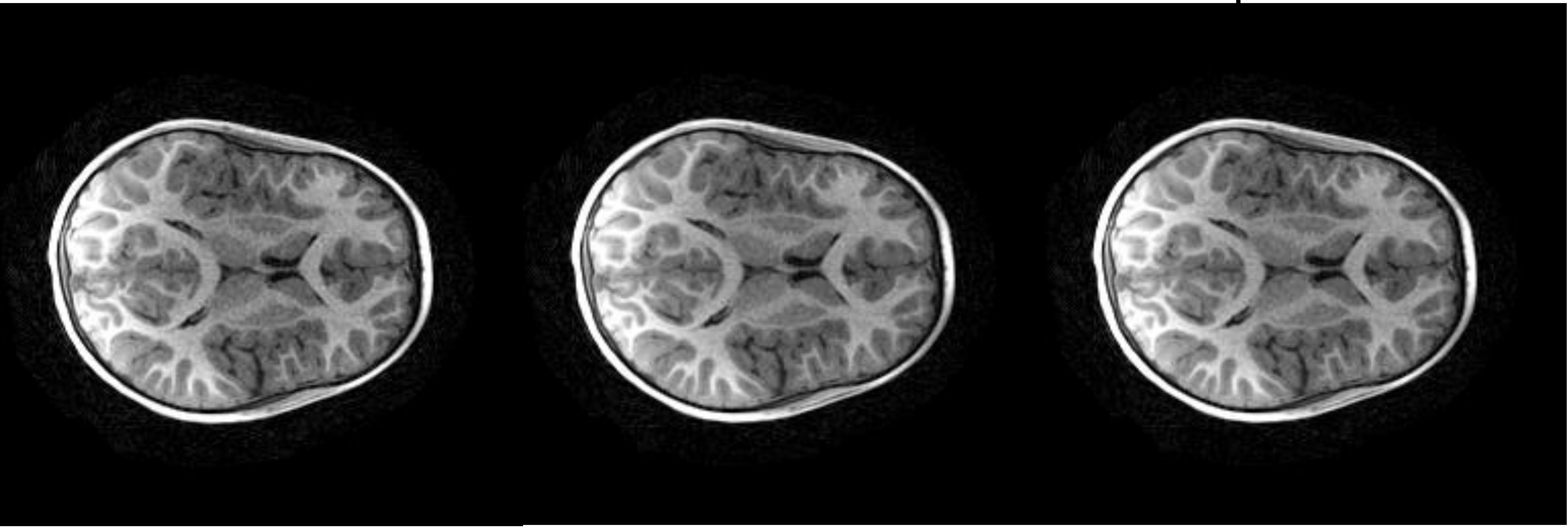

Jumpy

Fig. S28

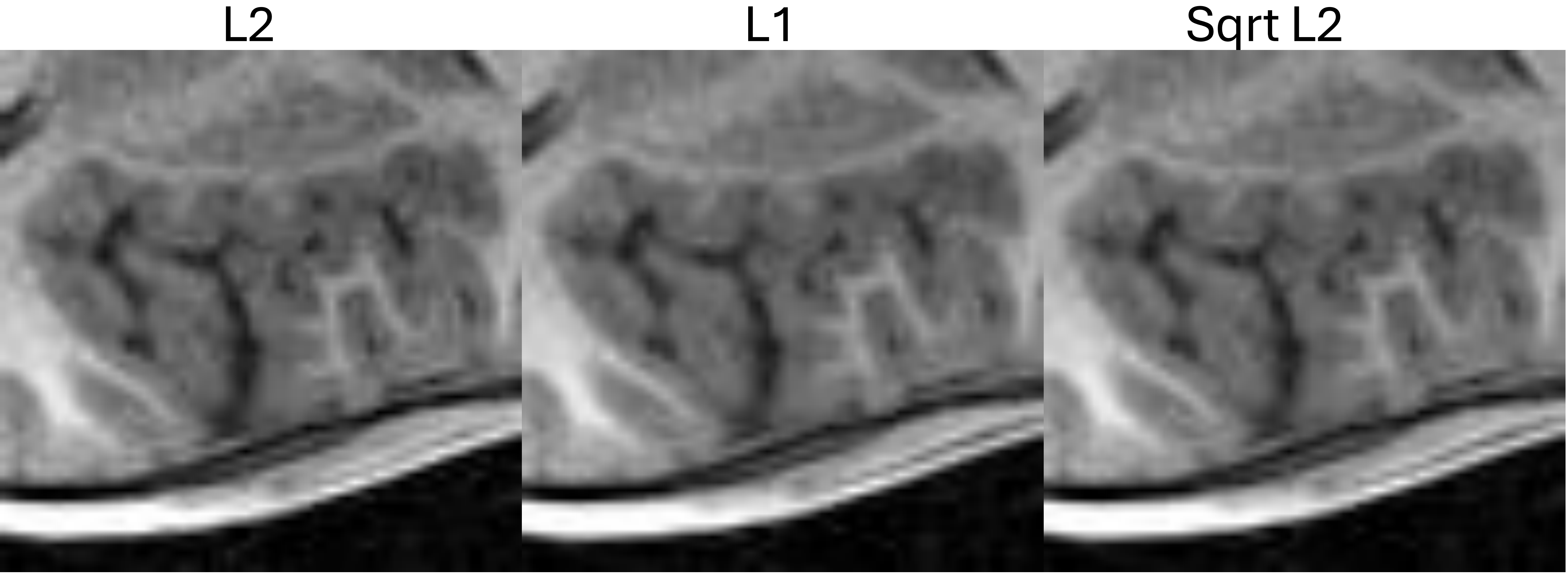

Same slice, zoomed in

Fig. S29

L2 L1 Sqrt L2

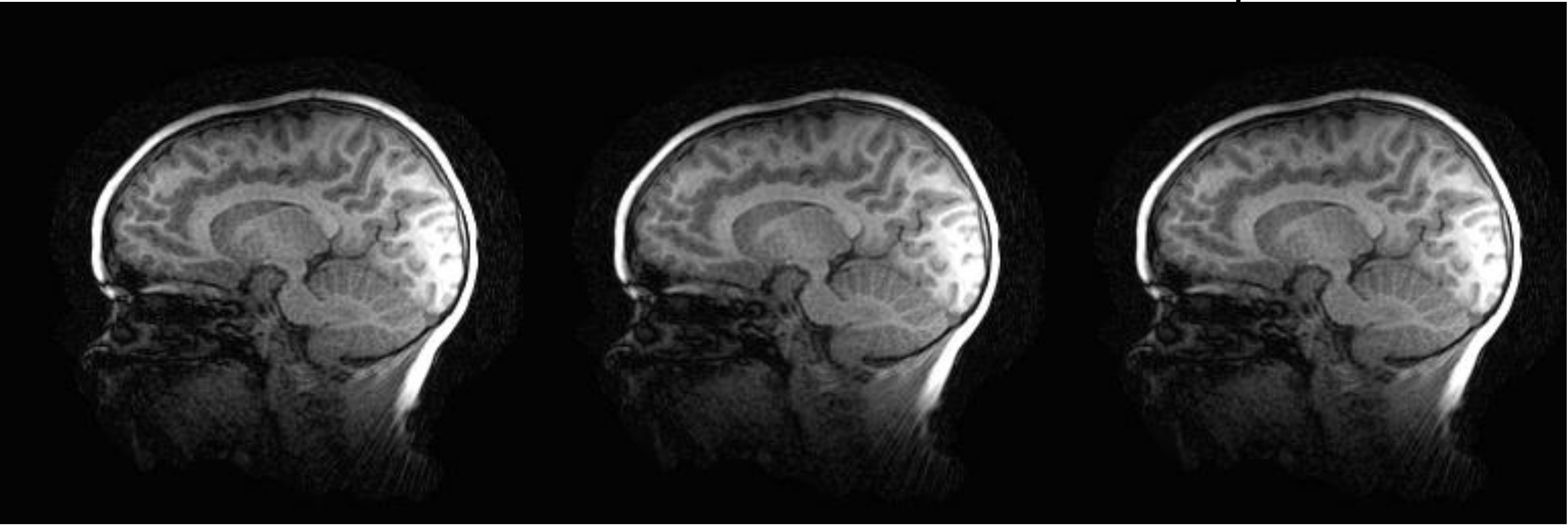

Jumpy

Fig. S30

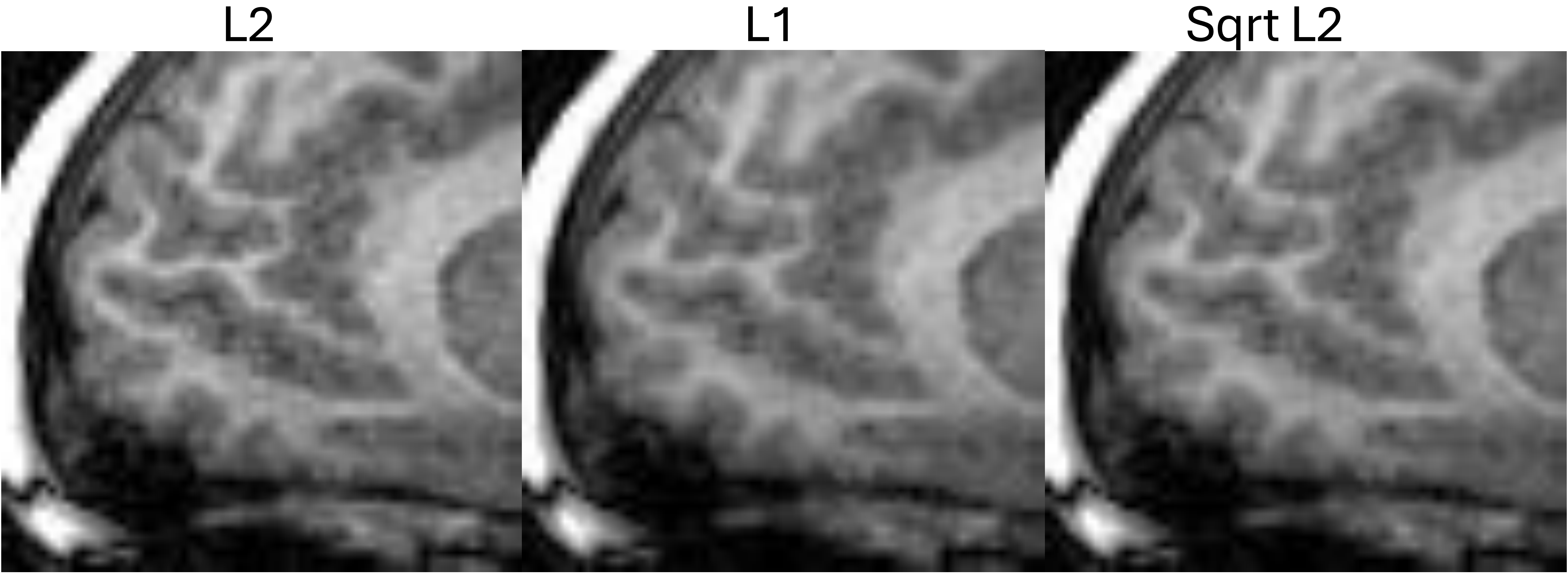

Same slice, zoomed in

Fig. S31

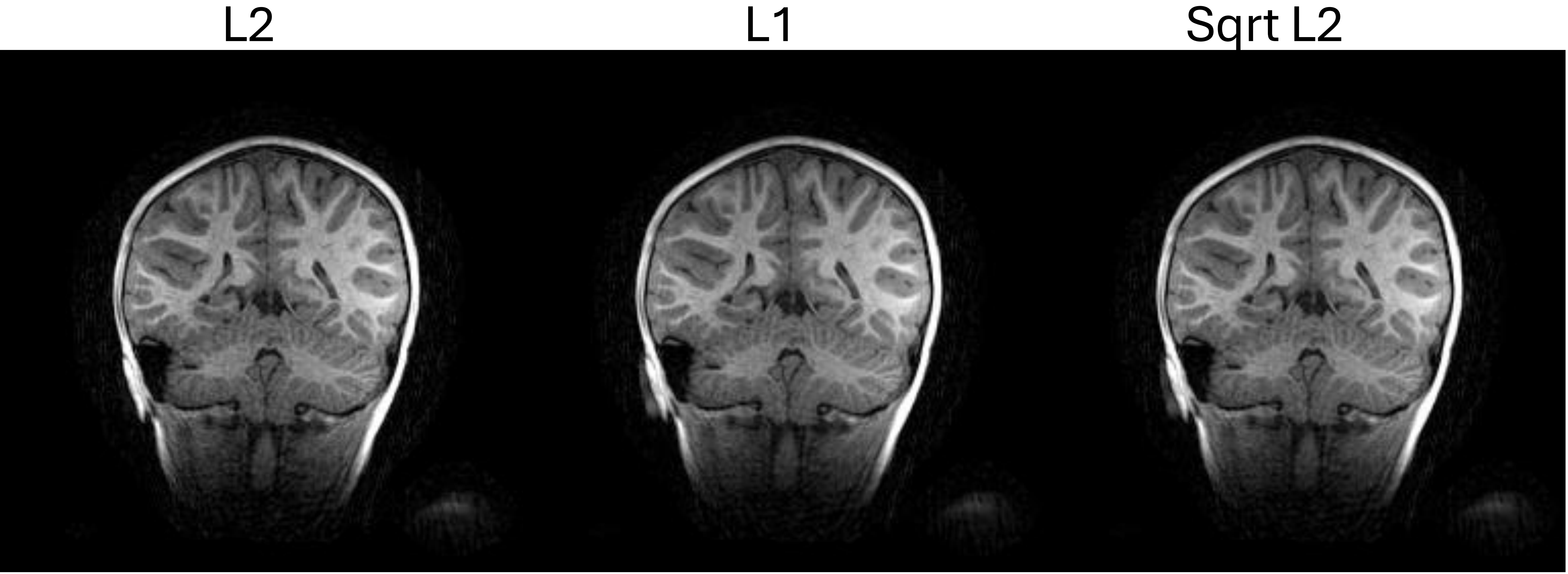

Jumpy

Fig. S32

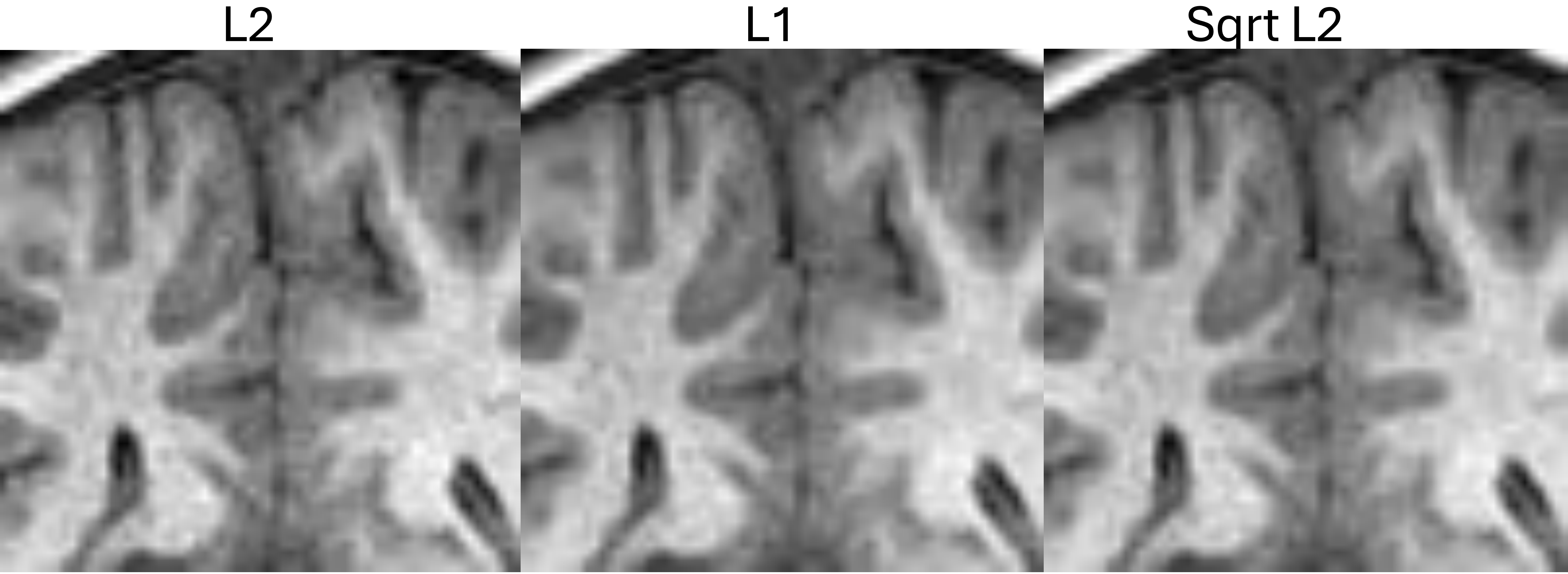

Same slice, zoomed in

Fig. S33

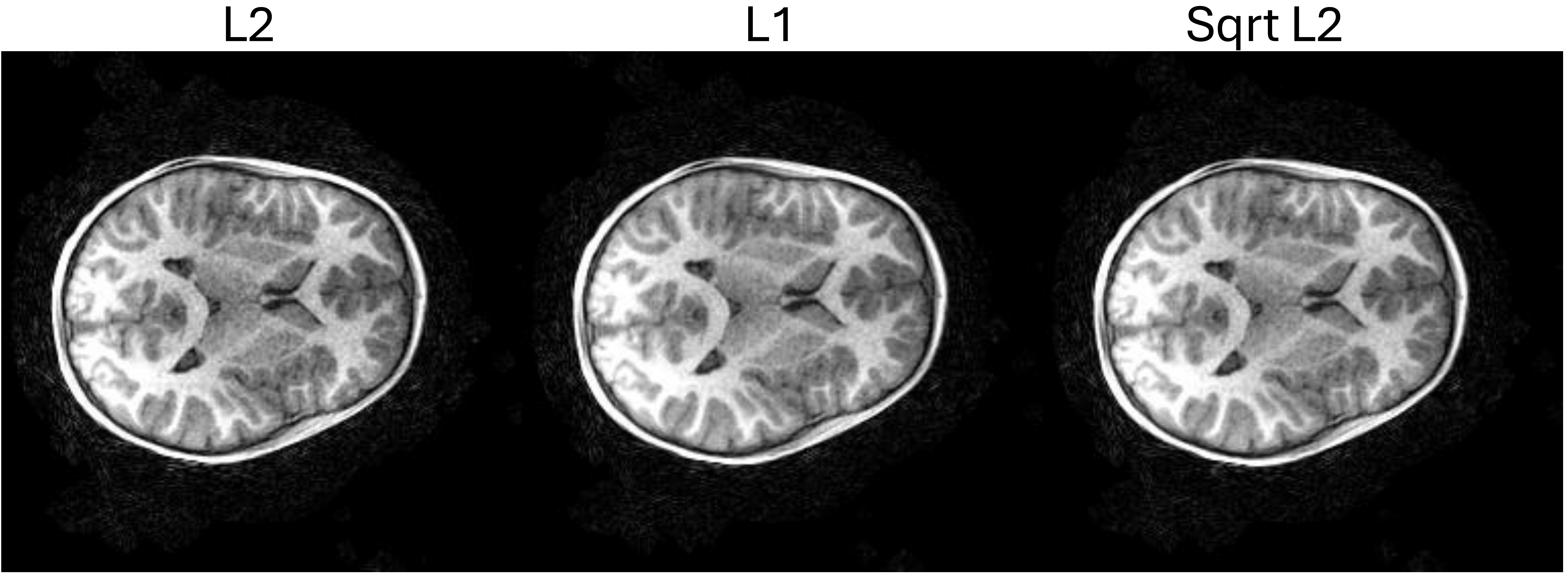

Severe

Fig. S34

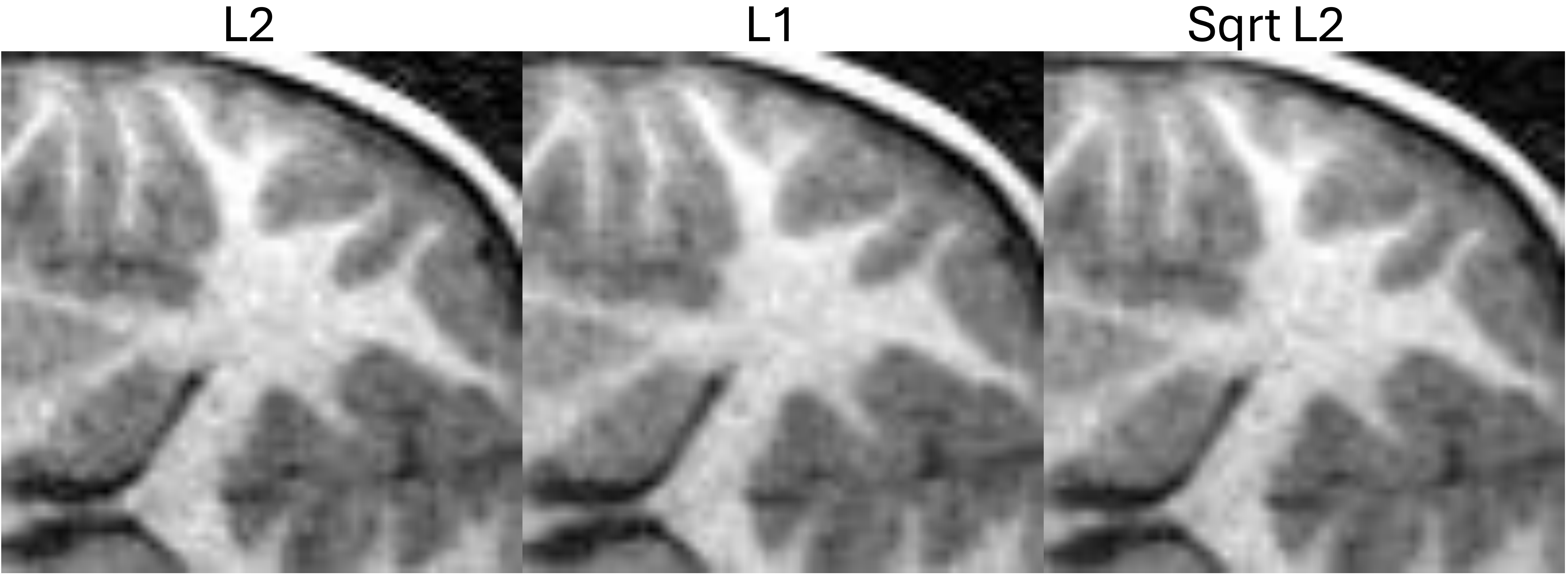

Severe

Fig. S35

L2 L1 Sqrt L2

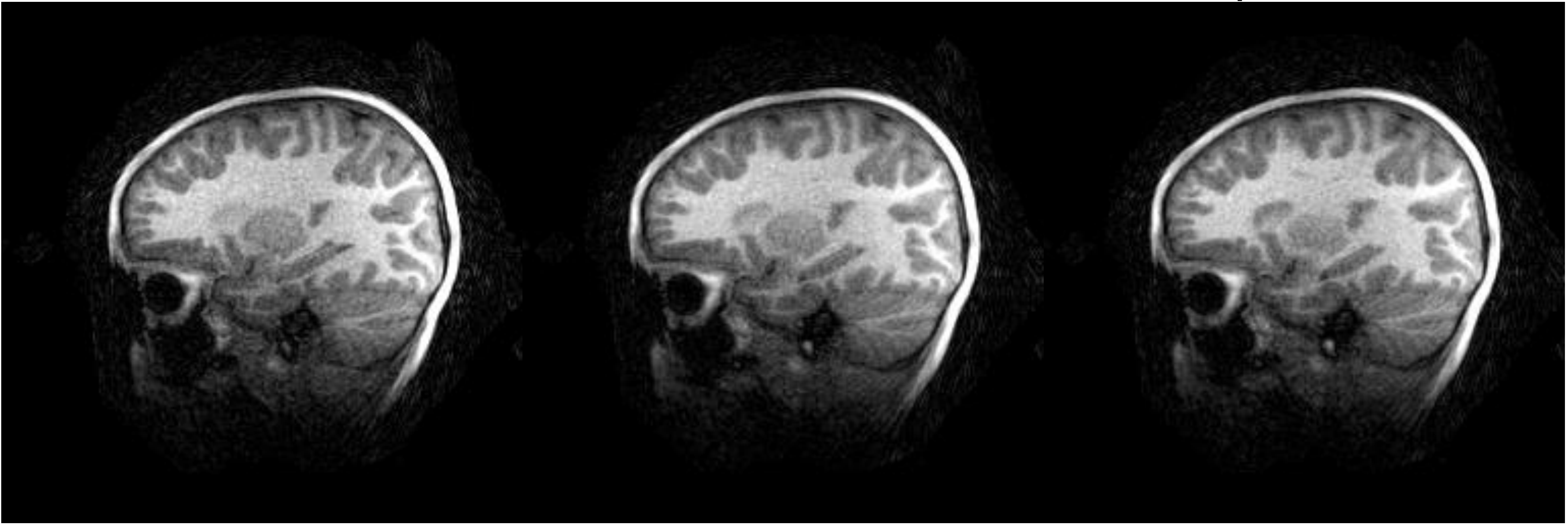

Severe

Fig. S36

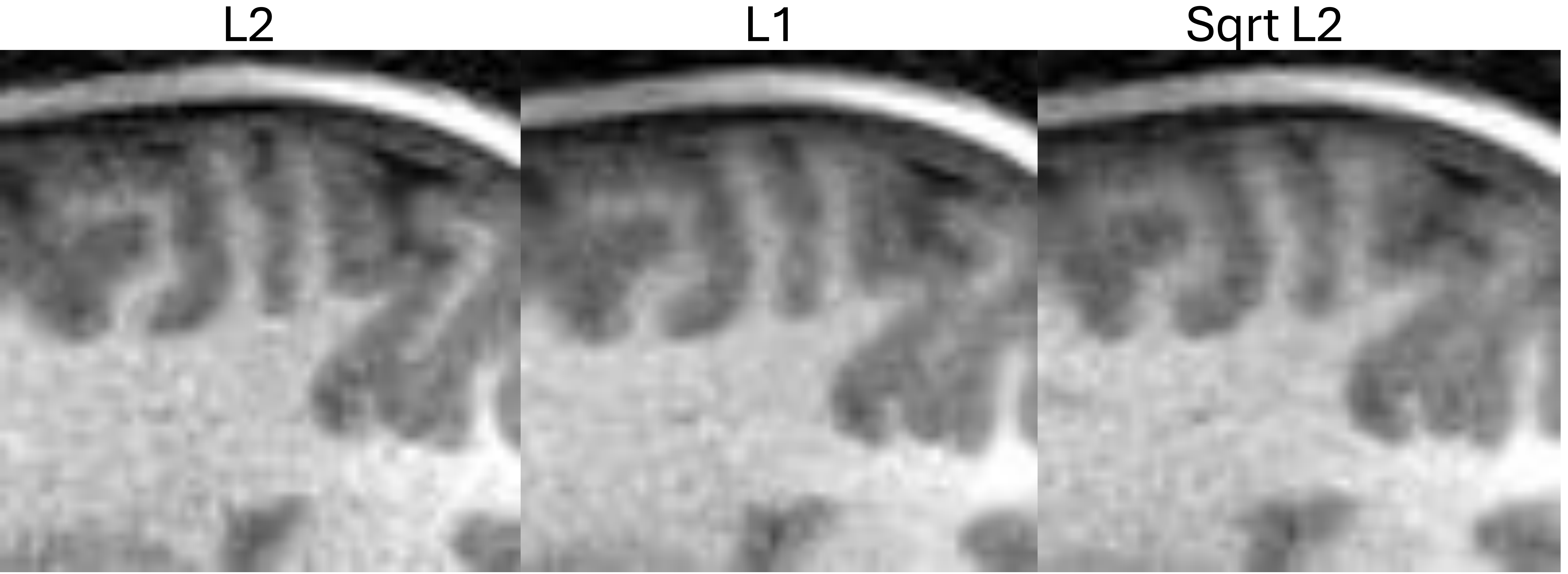

Same slice zoomed in

Fig. S37

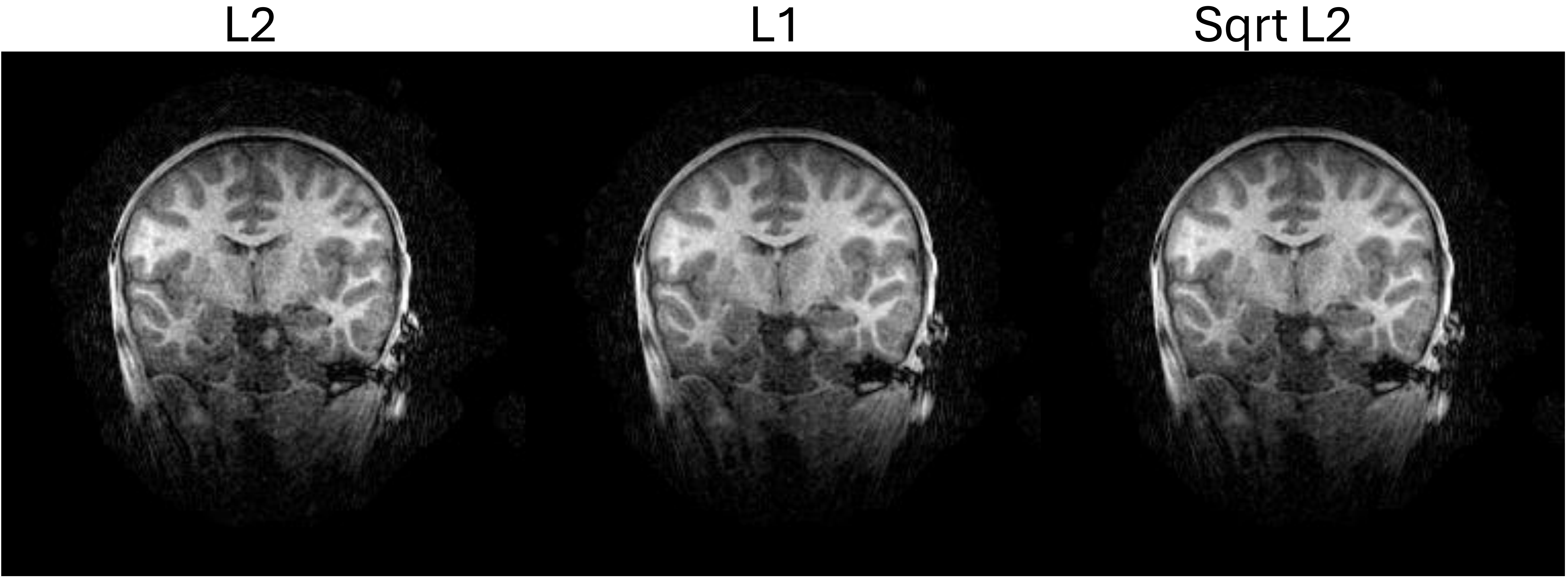

Severe

Fig. S38

L2 L1 Sqrt L2

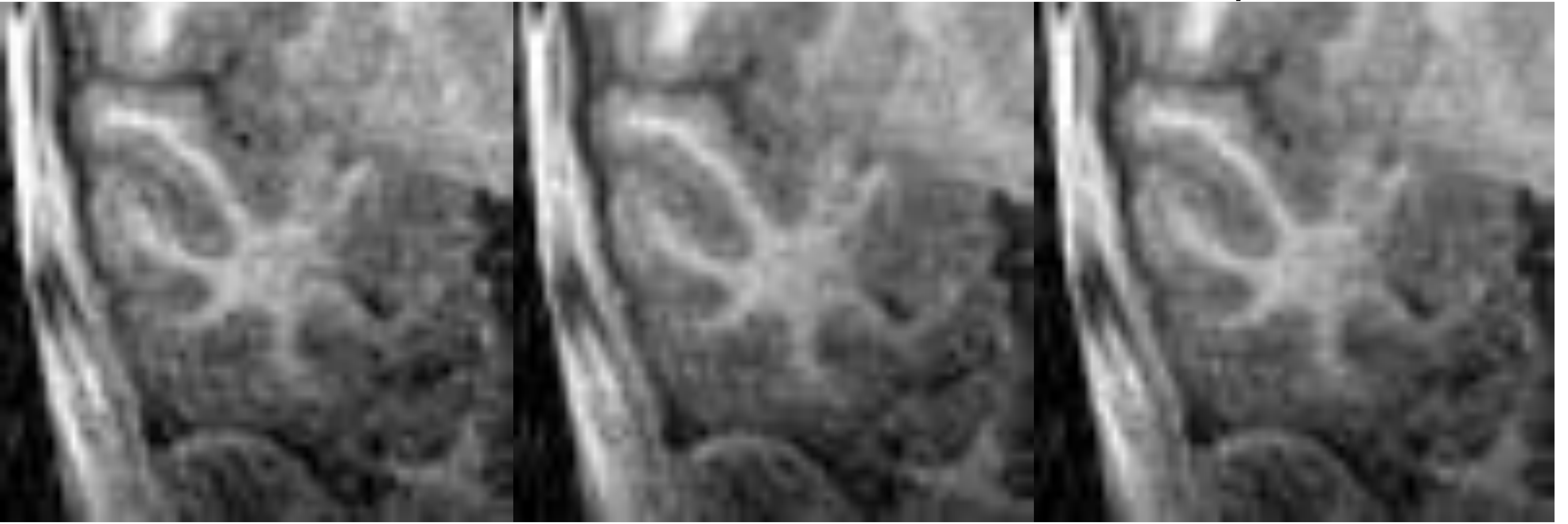

Same slice zoomed in

Fig. S39

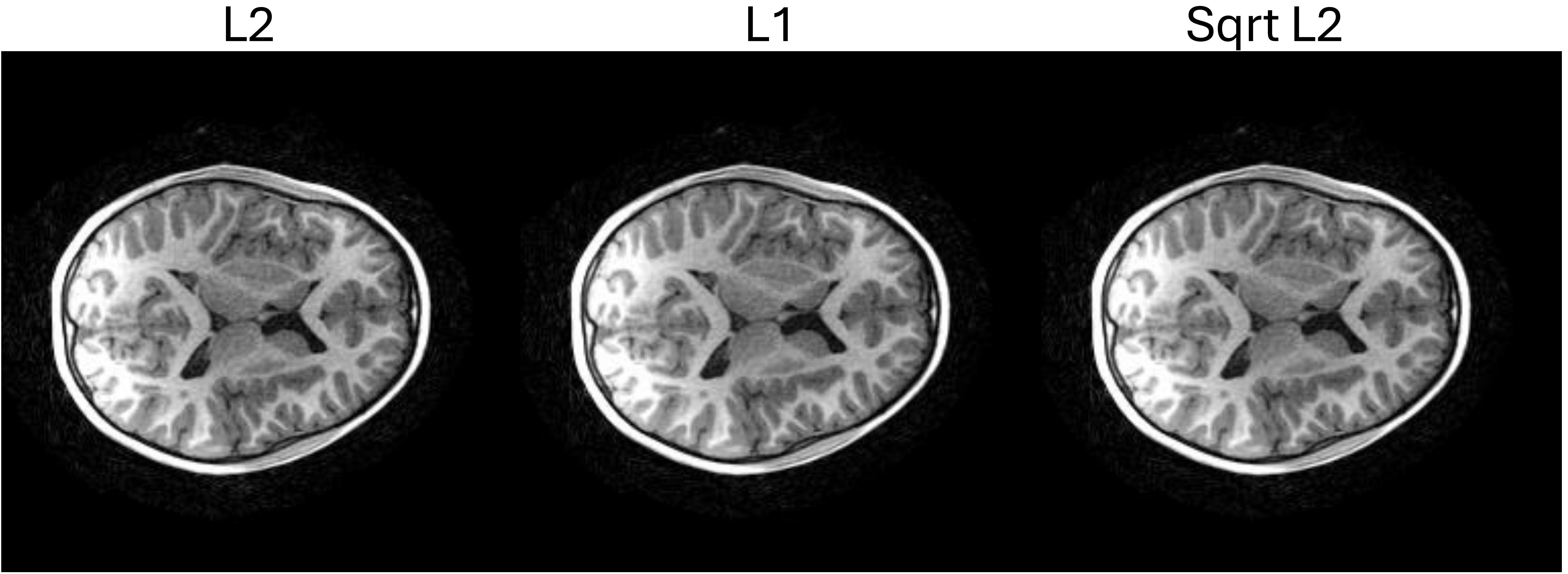

Jittery

Fig. S40

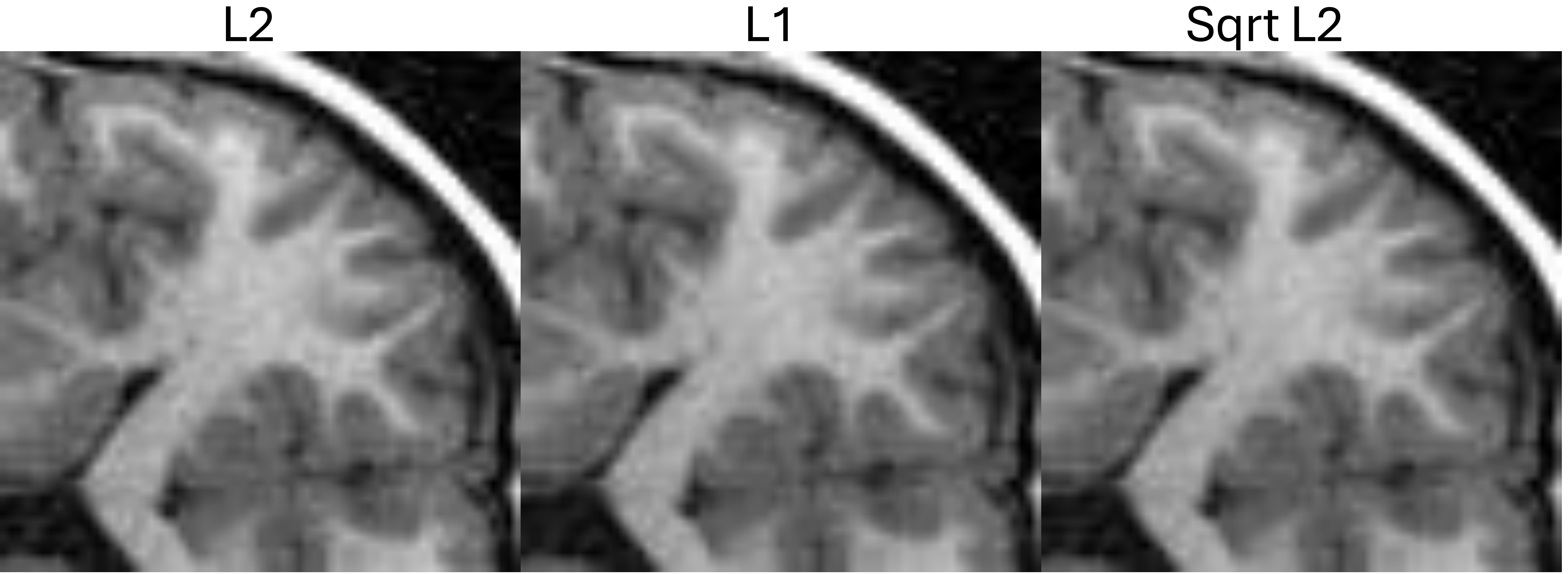

Same slice zoomed in

Fig. S41

L2 | L1 | Sqrt L2

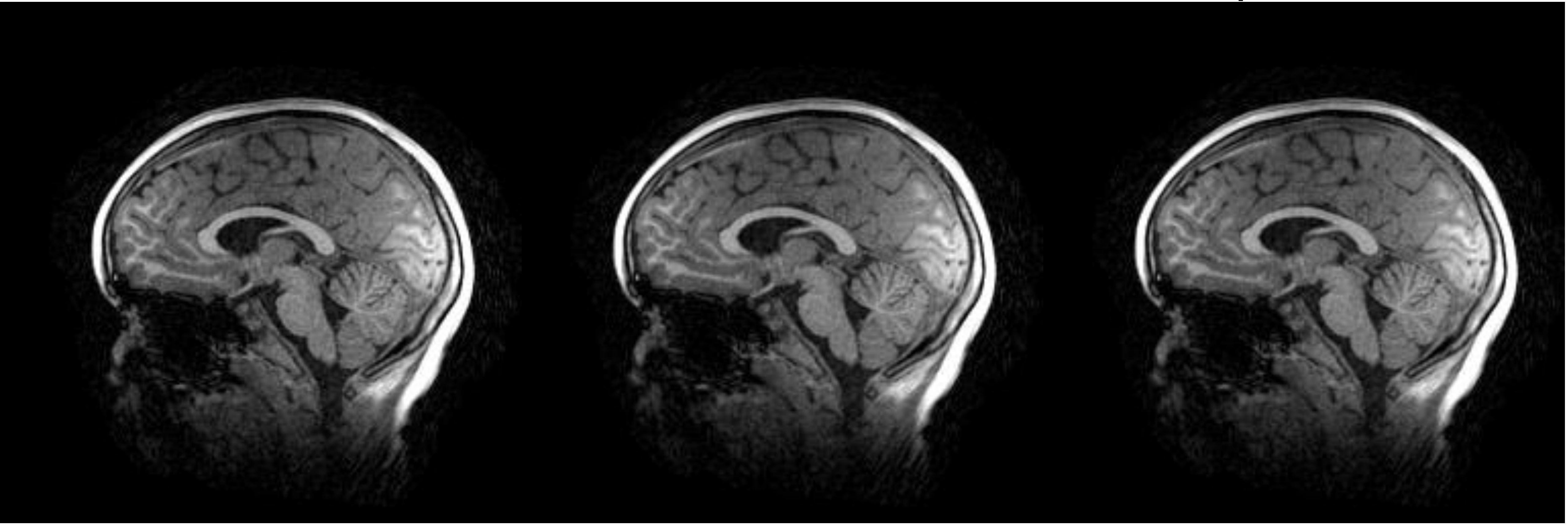

Jittery

Fig. S42

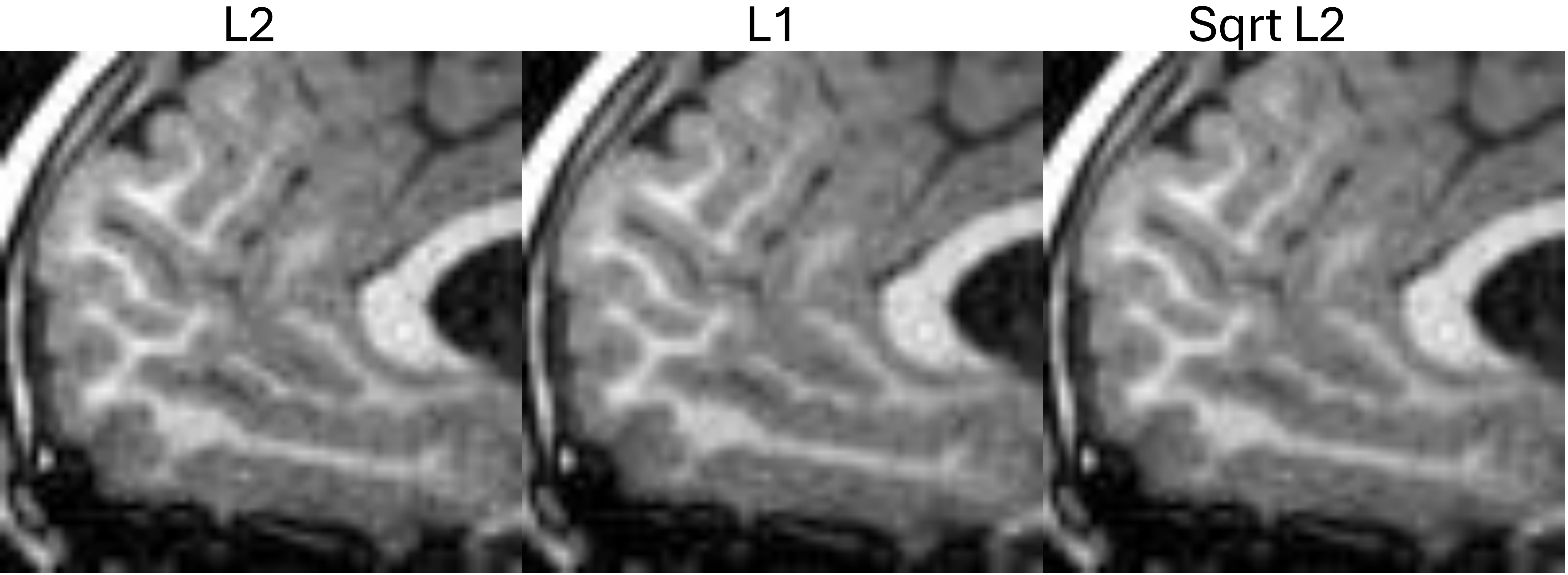

Same slice zoomed in

Fig. S43

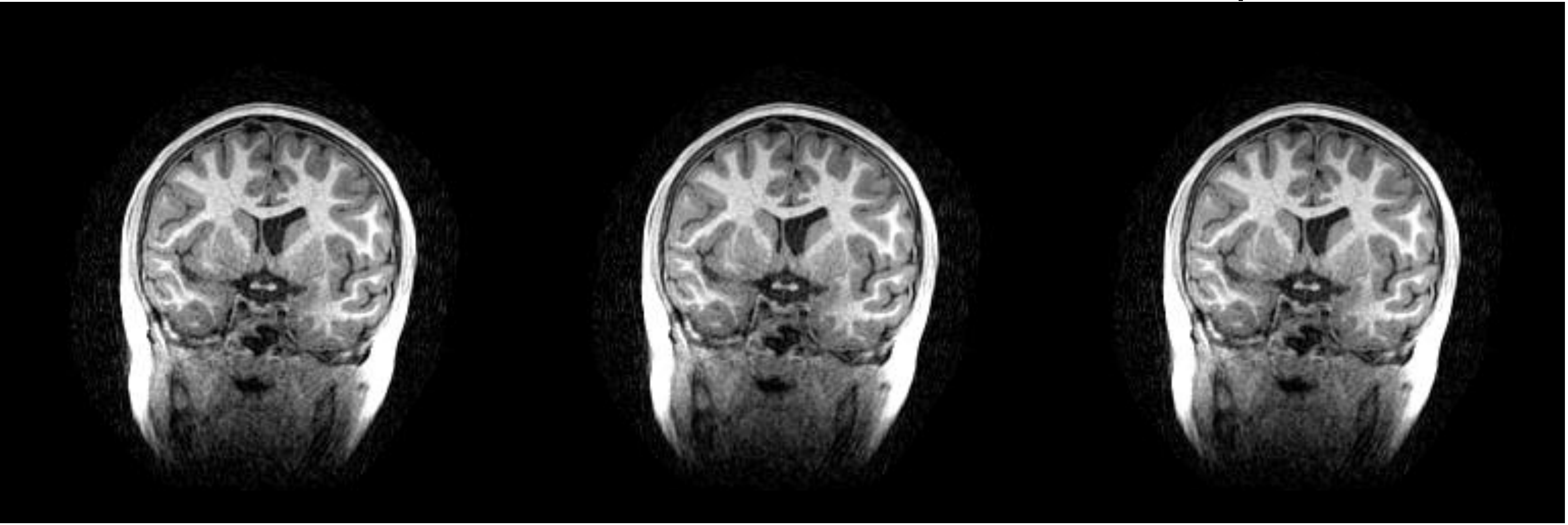

Jittery

Fig. S44

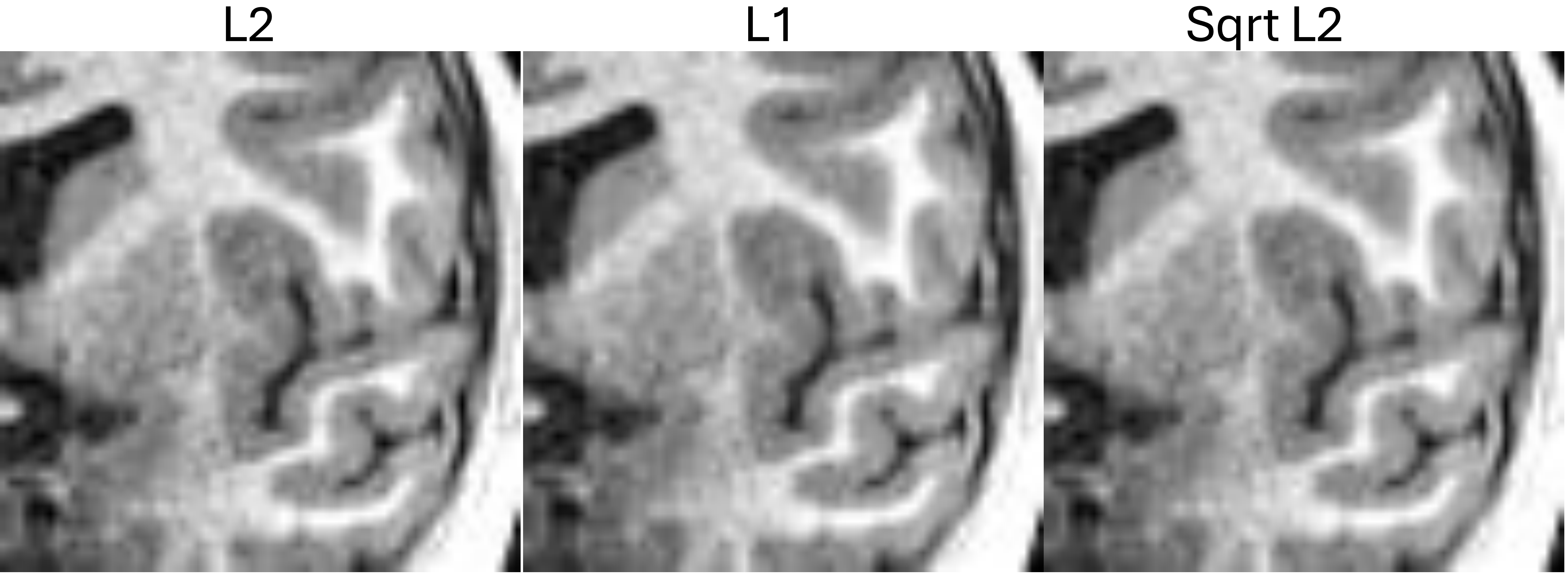

same slice zoomed in

Fig. S45
